# Understanding the impact of more realistic low-dose, prolonged engineered nanomaterial exposure on genotoxicity using 3D models of the human liver

**DOI:** 10.1186/s12951-021-00938-w

**Published:** 2021-06-28

**Authors:** Samantha V. Llewellyn, Gillian E. Conway, Ilaria Zanoni, Amalie Kofoed Jørgensen, Ume-Kulsoom Shah, Didem Ag Seleci, Johannes G. Keller, Jeong Won Kim, Wendel Wohlleben, Keld Alstrup Jensen, Anna Costa, Gareth J. S. Jenkins, Martin J. D. Clift, Shareen H. Doak

**Affiliations:** 1 In Vitro Toxicology Group, Institute of Life Science, Swansea University Medical School, Swansea University, Singleton Park, Swansea, SA2 8PP UK; 2 Advanced Materials Research, Department of Material Physics and Analytics, BASF SE, 67056 Ludwigshafen, Germany; 3 Advanced Materials Research, Department of Experimental Toxicology and Ecology, BASF SE, 67056 Ludwigshafen, Germany; 4 Institute of Science and Technology for Ceramics, CNR-ISTEC-National Research Council of Italy, Faenza, Italy; 5 National Research Centre for the Working Environment (NRCWE), Lersø Parkallé 105, 2100 Copenhagen, Denmark; 6 Korea Research Institute of Standards and Science (KRISS), 267 Gajeong-ro, Daejeon, 34113 Korea

**Keywords:** In vitro liver models, Engineered nanomaterials, Physiologically relevant exposure, Nanotoxicology, Genotoxicity

## Abstract

**Background:**

With the continued integration of engineered nanomaterials (ENMs) into everyday applications, it is important to understand their potential for inducing adverse human health effects. However, standard in vitro hazard characterisation approaches suffer limitations for evaluating ENM and so it is imperative to determine these potential hazards under more physiologically relevant and realistic exposure scenarios in target organ systems, to minimise the necessity for in vivo testing. The aim of this study was to determine if acute (24 h) and prolonged (120 h) exposures to five ENMs (TiO_2_, ZnO, Ag, BaSO_4_ and CeO_2_) would have a significantly different toxicological outcome (cytotoxicity, (pro-)inflammatory and genotoxic response) upon 3D human HepG2 liver spheroids. In addition, this study evaluated whether a more realistic, prolonged fractionated and repeated ENM dosing regime induces a significantly different toxicity outcome in liver spheroids as compared to a single, bolus prolonged exposure.

**Results:**

Whilst it was found that the five ENMs did not impede liver functionality (e.g. albumin and urea production), induce cytotoxicity or an IL-8 (pro-)inflammatory response, all were found to cause significant genotoxicity following acute exposure. Most statistically significant genotoxic responses were not dose-dependent, with the exception of TiO_2_. Interestingly, the DNA damage effects observed following acute exposures, were not mirrored in the prolonged exposures, where only 0.2–5.0 µg/mL of ZnO ENMs were found to elicit significant (*p* ≤ *0.05*) genotoxicity. When fractionated, repeated exposure regimes were performed with the test ENMs, no significant (*p* ≥ *0.05*) difference was observed when compared to the single, bolus exposure regime. There was < 5.0% cytotoxicity observed across all exposures, and the mean difference in IL-8 cytokine release and genotoxicity between exposure regimes was 3.425 pg/mL and 0.181%, respectively.

**Conclusion:**

In conclusion, whilst there was no difference between a single, bolus or fractionated, repeated ENM prolonged exposure regimes upon the toxicological output of 3D HepG2 liver spheroids, there was a difference between acute and prolonged exposures. This study highlights the importance of evaluating more realistic ENM exposures, thereby providing a future in vitro approach to better support ENM hazard assessment in a routine and easily accessible manner.
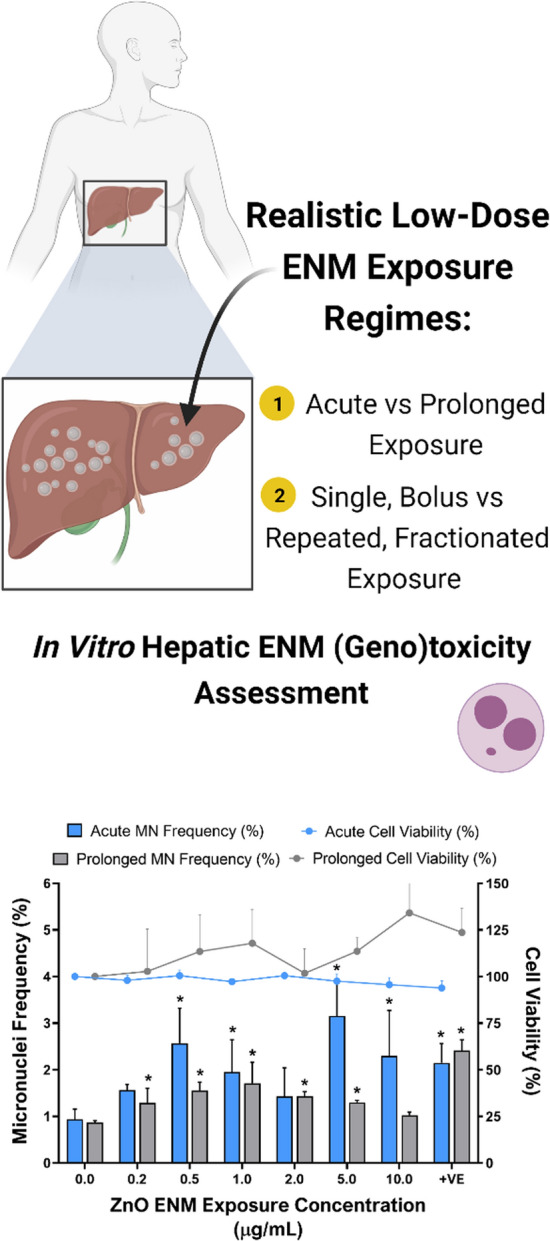

**Supplementary Information:**

The online version contains supplementary material available at 10.1186/s12951-021-00938-w.

## Background

Nanotechnology is considered an important Key Enabling Technology (KET), underpinning a variety of novel applications across wide ranging sectors. As a global market, nanotechnology reached $75.8 billion in 2020 and is predicted to exceed $125 billion in the next three years, with engineered nanomaterials (ENMs) defined as having the greatest share of the global nanotechnology market [1]. ENMs are manufactured materials with advanced size specific physico-chemical properties derived from an unbound, monodispersed state, or as an aggregate/agglomerate where 50% or more of the particles possess one or more external dimensions in the size range 1–100 nm [2]. This greater surface area to volume ratio enables ENMs to harbour advantageous properties that improve the functionality of a plethora of applications (e.g. cosmetics, medicine, electronics, construction and energy industries) providing great opportunities for economic growth and life improving technologies. Consequently, with increasing human and environmental exposure comes the need to understand any potential associated safety risks.

Human ENM exposure occurs via four primary routes; inhalation, ingestion, injection and dermal penetration. With the exception of certain medical treatments, the prospect of injecting ENMs into the body is relatively low for the majority of individuals. While for most ENM, the likelihood of occupational inhalation exposure is predominant, such as the use of barium sulfate (BaSO_4_) and cerium dioxide (CeO_2_) in the automotive industry; other routes of potential relevant exposure could arise from the use of consumer products, with some examples being the ingestion of food grade titanium dioxide (TiO_2_) or dermal penetration of sunscreen enhancing zinc oxide (ZnO) [3–6]. Silver (Ag) ENMs, with its popular anti-microbial properties, are deemed the most readily applied ENM in consumer products included in the top three applications found in medicine, textiles and cosmetic products [7–9]. Consequently, understanding the impact of repeated ENM exposure to human health over prolonged periods of time is imperative.

Once ENMs have entered the body, if they have the ability to traverse biological barriers and enter circulation, the materials can translocate to secondary sites of deposition, including the spleen, liver and kidneys [10, 11]. Of these sites, the liver is of particular toxicological importance due to its high susceptibility to ENM deposition and accumulation, as well as its role in maintaining metabolic homeostasis and the detoxification of both endogenous and exogenous substances [12, 13]. Secondary ENM deposition in the liver is commonly reported, but it is becoming more evident that translocation following inhalation or ingestion in particular, is low with < 1.0% of the insoluble ENM exposure concentration reaching the secondary organs [14]. A previous in vivo study undertaken to assess the effects of an occupational 14-day pulmonary exposure, found that only 1.24% and 2.87% of the original intratracheal instilled dose of 162 µg of TiO_2_ and CeO_2_ ENMs per mouse reached the liver [15]. This corresponds to a translocated dose of 1 µg/g in vivo or 1 µg/mL in vitro [15], illustrating the necessity for low-dose exposures when evaluating the effect of ENM exposure upon secondary sites of exposure, such as the liver. Even at low-doses, ENMs have been found to induce hepatic dysfunction and severe organ damage in vivo. Liver damage caused by the long-term (90 days), daily intragastric exposure of 2.5–10 µg/g of TiO_2_ resulted in bioaccumulation and aggregation in the liver over time, significant changes in tissue morphology and the expression of genes involved in immune and inflammatory responses (e.g. CXCII over-expression), apoptosis, oxidative stress and metabolic process [16]. Similarly, low dose (< 10.0 µg/mL) in vitro ENM exposures of Ag and ZnO were sufficient to induce biological effects, including DNA damage and elevated levels of reactive oxygen species (ROS). Yet, many in vitro studies have previously focused on the acute effects of high ENM concentrations, which is beneficial for establishing potential toxicity and hazard, but does not allow for evaluation of the chronic effects that may be associated with the more realistic long-term human exposure scenarios [14, 17]. Prolonged exposure to ENMs may induce repetitive injury leading to chronic liver disease, whereby the regenerative capabilities are impaired, and the hepatocytes begin to undergo cell death as a result of inflammation [12, 18]. Therefore, continued ENM exposure raises concerns regarding the gradual accumulation and chronic health effects that may be induced.

With the vast range of ENMs available on the market, each with its own unique specification, it is untenable to rely on in vivo based methods to fully elucidate the immediate and lasting effects of ENM exposure upon the human body [19]. In recent years, international bodies such as the Organisation for Economic Cooperation and Development (OECD) Working Party on Manufactured Nanomaterials, the International Organization for Standardization (ISO) and the European Committee for Standardization (CEN) have published a series of test guidelines, guidance documents and regulatory standards to help drive the development of physiologically relevant, high-throughput in vitro test systems and regulated protocols for ENM hazard assessment [20]. To align with these new test guidelines and the 3Rs directive to replace, reduce and refine the use of in vivo based testing systems, researchers have been developing advanced in vitro models to emulate human organ systems and sustain long-term culture, to provide viable alternatives to in vivo test systems and to propel in vitro ENM hazard assessment screening forward.

The development of 3D liver models has been an important advancement as they have been shown to better encapsulate organ morphology and intricate multi-cellular interactions, while demonstrating improved hepatic function, metabolic activity, and extended culture longevity [12, 21–24]. To date, the longest viable hepatic model in vitro is described to be functional for up to 5 months with the use of an inverted colloidal crystal extracellular matrix (ECM) to aid the formation of hepatic hexagonal architecture with primary human foetal hepatocytes [25]. Though this model has the capability to parallel in vivo based long-term exposure studies, the use of an ECM scaffold, however, poses different challenges for ENM hazard assessment [26, 27]. Some 3D hepatic in vitro models based on primary human hepatocytes (PHH) can remain viable for up to 21 days in culture; whilst they can be utilised for the evaluation of e.g. viability, hepatic functionality, metabolic activity and pro-inflammatory response, they do not actively proliferate and so cannot be used for genotoxicity testing which often requires proliferating cells [14]. To overcome this, a proliferating cell-line based 3D in vitro HepG2 spheroid model has been developed, which can be utilised to evaluate multiple toxicological endpoints including genotoxicity [28, 29]. HepG2 cells have shown phenotypic secretion of hepatic plasma proteins (e.g. albumin, fibrinogen and transferrin), phase II gene expression and the capability to positively identify pro-carcinogens and six out of nine drug-induced liver injury (DILI) compounds [24, 30, 31].

Given the limitations associated with standard in vitro hazard testing approaches, this study aimed to determine if more realistic prolonged and repeated ENM exposure regimes exhibited different (geno)toxicological outcomes as compared to standard acute exposures when utilising 3D liver spheroids. Five ENMs were selected based on the ECETOC DF4nanoGrouping decision-making framework for the grouping and testing of ENM, to provide a range of materials that possess different physico-chemical characteristics and exhibit varying dissolution and transformation capacities in a biological environment; TiO_2_ and CeO_2_ defined as active, insoluble materials, ZnO and Ag defined as soluble, ionic materials and BaSO_4_ defined as a passive, non-reactive material [32, 33]. Furthermore, this study sought to determine the genotoxic potency of these materials, at physiologically relevant, low exposure concentrations of 0.2–10.0 µg/mL, upon the 3D in vitro liver models.

## Results

### ENM intrinsic and extrinsic physico-chemical characterisation

Deemed as the defining features responsible for the beneficial integration of ENMs into various applications and the drivers of ENM toxicity, the characterisation of individual ENM physico-chemical properties is crucial in understanding the interaction, uptake, translocation and potential adverse effects of these materials within biological systems. Five different metallic ENMs were evaluated using physico-chemical characterisation techniques to assess ENM composition, crystallinity, size, surface area, surface properties (e.g. coating, charge, reactivity), solubility, dissolution and bio-persistence.

A summary of all the intrinsic and extrinsic physico-chemical characteristics of the test ENMs are provided in Table 1. The five materials, whilst composed of varying metals, share a similar size and density of 10–50 nm in diameter and 3.5–8.5 g/cm^3^, respectively. All the ENMs exhibit a similar primary particle size, but they have different specific surface areas (as measured by BET) with Ag possessing the lowest specific surface area of 6.4 m^2^/g and TiO_2_ possessing the highest specific surface area of 51.0 m^2^/g. All the ENMs have a negative surface charge at pH 7 in 10 mM of KCl water solution, with the exception of CeO_2_ with a positive surface charge of +35.2 mV. Only two ENMs, Ag and ZnO, both of which are commonly found to dissociate into ions, have a surface coating; functionalized PVP and UV activated silicon, respectively. Further to this, according to the definitions set out by Arts et al., they are the only two ENMs tested in this study that are hydrophobic, with a water contact angle >90°. Yet, they were both originally categorized as ‘soluble, non-persistent ENMs’ by the ECETOC DF4nanoGrouping decision-making framework, pertaining to the high rates of dissolution observed [32, 33].

**Table 1:** Physico-chemical characteristics of TiO_2_, ZnO, Ag, BaSO_4_ and CeO_2_ ENMs

ENM physico-chemical characterisation	TiO_2_ NM-105	ZnO NM-111	Ag Sigma 576832	BaSO_4_ NM-220	CeO_2_ NM-212
Core composition and CAS	Titanium Dioxide CAS: 1317-80-2	Zinc Oxide CAS: 1314-13-2	Silver CAS: 7440-22-4	Barium Sulfate CAS: 7727-43-7	Cerium Dioxide CAS: 1306-38-3
Crystalline phases (XRD)^a^	86.9% Anatase + 13.1% Rutile	Zincite	Metallic	Barite	Cerianite
Surface coating	N/A	UV active silicon coating—triethoxycaprylsilane	PVP (polyvinylpyrrolidone) functionalized polymer	N/A	N/A
Impurities (XRF)	None (purity > 99%)	P_2_O_5_, SiO_2_, CaO, CuO, Fe_2_O_3_, NiO (All < 1.0%)	Pd, Cl (< 1.0%) Rh, Fe, Cu, Ni (< 0.1%) (purity > 99.5%)	Na, Ca, Sr, F, Cl, organic contaminations (purity > 93.8%)	P_2_O_5_, CaO, Cl (< 1.0%) V_2_O_5_, SO_3_, CoO, MgO, SiO_2_, CuO, Fe_2_O_3_, ZnO (< 0.1%)
Size (MinFeret; TEM)^b^	25 nm	40.6 nm	30.0 nm	31.5 nm	13.7 nm
3D aspect ratio and circulatory (TEM)^b^	N/A	A.R: 1.88 ± 0.78 C: 0.80 ± 0.12	A.R: 1.36 ± 0.30 C: 0.88 ± 0.09	A.R: 1.22 ± 0.19 C: 0.98 ± 0.04	A.R: 1.21 ± 0.25 C: 0.97 ± 0.06
Surface area (BET)	51.0 m^2^/g	12.0 m^2^/g	6.4 m^2^/g	33.0 m^2^/g	27.0 m^2^/g
Relative density (He pycnometer)	3.95 g/cm^3^	4.99 g/cm^3^	8.36 g/cm^3^	4.13 g/cm^3^	7.2 g/cm^3^
Chemical nature of the surface (XPS)^c^	Ti: 24.5% O: 65.0% C: 10.5%	Zn: 34.6% C: 22.2% O: 43.1%	Ag: 38.9% C: 47.6% O: 13.5%	Ba: 21.5% S: 12.5% O: 65.8%	Ce: 25.6% O: 74.4%
Surface charge (zeta potential at pH 7 in 10 mM KCl water solution)	− 17.0 mv	N/A	− 30.0 mV	− 30.2 mV	+ 35.2 mV
Surface reactivity (FRAS)	18.6 (nmol TEU/m^2^ ENM)	20.3 (nmol TEU/m^2^ ENM)	2240.0 (nmol TEU/m^2^ ENM)	13.9 (nmol TEU/m^2^ ENM)	16.7 (nmol TEU/m^2^ ENM)
Surface reactivity (EPR DMPO and CPH spin count)	DMPO: 3.01e^+12^ CPH: 8.29e^+12^	DMPO: 1.21e^+13^ CPH: 1.28e^+13^	DMPO: 8.00e^+11^ CPH: 5.58e^+13^	DMPO: 4.92e^+12^ CPH: 8.89e^+12^	DMPO: 5.86e^+12^ CPH: 1.22e^+14^
Hydrophobicity (water contact angle)	Hydrophilic (60°)	Hydrophobic (152°)	Hydrophobic (141°)	Hydrophilic (< 10°)	Hydrophilic (60°)
Dissolution^d^	Low dissolution (in PBS)	High dissolution (in biological media DMEM + FCS)	Unknown	Low dissolution (in PBS)	Low dissolution (in biological media DMEM + FCS)
Dissolution (24 h) in DMEM + 10% FBS + 1% Pen/Strep	Not detectable	10.11 ± 0.31 µg/mL	0.01 ± 0.01 µg/mL	2.40 ± 0.01 µg/mL	Not detectable
Biological clearance in vivo (t50)^d^	Physiological clearance (> 40 days)	Rapid clearance	Unknown	Accelerated clearance (< 40 days)	Decelerated clearance (> 40 days)
Previous REACH grouping category^d^	4—active, biopersistent, non-fibrous ENMs	1—soluble, non-persistent ENMs	1—soluble, non-persistent ENMs	3—passive, biopersistent, non-fibrous ENM	4—active, biopersistent, non-fibrous ENMs

Characterisation techniques are abbreviated, in order of appearance, as follows: *CAS* chemical abstracts service, *XRD* X-ray diffraction, *XRF* X-ray fluorescence, *TEM* transmission electron microscopy, *BET* Brunauer–Emmett–Teller, *XPS* X-ray photoelectron spectroscopy, *FRAS* ferric reduction ability of serum, *EPR* electron paramagnetic resonance spectroscopy, *DMPO* 5,5-dimethyl-1-pyrroline-*N*-oxide, *CPH* 1-hydroxy-3-carboxy-pyrrolidine, *REACH* registration, evaluation, authorisation and restriction of chemicals

All data indicated with a ^a^is complemented by TEM images, sourced from Keller et al. and Yin et al. ^b^is complemented by XRD graphs ^c^is complemented by XPS graphs located in the Additional file 1: Figure S1, S2 and S3, respectively. All data with a ^d^was sourced from Arts et al. 2016 [32, 34, 35]

The colloidal behaviour of the five ENMs in the test medium (DMEM) over a period of 24 and 120 h was determined using dynamic light scattering (DLS), as shown in Table 2. In conjunction, the polydispersity index (PDI; measure of ENM sample heterogeneity based on size), and zeta potential (ZP; surface charge), of the materials was measured. For TiO_2_, we observed a concentration dependent increase in size distribution, from ~20 nm (0.2 µg/mL) to 300 nm (10 µg/mL). We found comparable results in the size distribution after 24 and 120 h exposure that confirms the ability of complete DMEM medium to preserve colloidal stability of TiO_2_ ENMs, even after prolonged exposure. However, all the samples showed high PDI values, presenting polydispersion in the size distribution (3 main populations were detected at all concentrations). The ZP data was set around −10 mV which aligns the with ZP value of complete DMEM and confirms the presence of a uniform protein corona surrounding the ENMs at all exposure concentrations and time points. The only difference detected with ZnO ENMs in complete DMEM, in comparison with TiO_2_, is that the size remained below 50 nm for all the samples tested and thus do not exhibit a tendency to actively agglomerate. As for TiO_2_, the ZnO ZP values do not significantly change as a function of time and exposure concentration, remaining within 0 to −10 mV, illustrating little surface charge to encourage agglomeration. In Ag samples, after 24 h, the samples showed a broad distribution, with an abrupt increase in diameter for more concentrated samples (2.0–10.0 µg/mL). However, after 120 h, the particle size distribution was narrower and the mean size values are reduced (1.0–10.0 µg/mL samples). Also, in this case, the most reliable hypothesis is that the larger diameter particles sedimented and the complete DMEM stabilized the nano-fraction left in suspension. BaSO_4_ ENMs appear very small and exhibit a narrow size distribution for all the samples, resulting in a standard deviation around a few nanometers. The ENM agglomerate size almost doubles from 0.2 to 10.0 µg/mL at both time points, but this material demonstrated the greatest stability and dispersion. In a similarly manner to TiO_2_, CeO_2_ ENMs displayed an increase in size distribution correlated with an increase in the exposure concentration. However, following 120 h exposure, the difference in size data between the lower and higher concentrations were very low. In fact, even if there is a 2-fold increase in CeO_2_ concentration, the mean diameter only slightly increases in size and retains a narrow distribution. Whilst this behaviour is indicative of an increase in colloidal stability versus time, it is more likely due to a partial sedimentation of larger particles that reduce particle size distribution over time.

**Table 2:** The colloidal behaviour of TiO_2_, ZnO, Ag, BaSO_4_ and CeO_2_ ENMs once exposed to DMEM complete media for 24 and 120 h.

ENM sample	ENM concentration (µg/mL)	Exposure time (h)	pH	Size DLS (nm)		PDI	Zeta Pot. (mV)	
				Mean	Stdev		Mean	Stdev
TiO_2_ NM 105	0.2	24	7.6	23	1	0.6	− 8.8	0.6
	0.5	24	7.7	108	64	0.3	− 10.2	0.6
	1	24	7.6	112	49	0.4	− 9.9	0.4
	2	24	7.7	226	46	0.3	− 8.0	0.4
	5	24	7.7	216	24	0.7	− 10.3	0.8
	10	24	7.6	282	22	0.6	− 9.8	0.6
	0.2	120	7.8	37	2	0.6	− 5.9	0.5
	0.5	120	7.8	97	21	0.3	− 10.0	0.8
	1	120	7.9	71	2	1.0	− 10.2	0.3
	2	120	7.9	232	24	0.5	− 13.1	0.9
	5	120	8.4	152	10	0.5	− 12.6	1.1
	10	120	8.0	275	10	0.4	− 10.1	0.7
ZnO NM 111	0.2	24	7.6	21	3	0.4	− 7.8	0.9
	0.5	24	7.7	25	3	0.6	− 6.7	1.6
	1	24	7.7	22	4	0.4	− 4.3	0.8
	2	24	7.7	20	1	0.4	− 7.7	0.5
	5	24	7.7	50	12	0.4	− 4.4	0.9
	10	24	7.7	74	43	0.2	− 8.3	0.8
	0.2	120	7.8	30	1	0.5	− 7.5	0.8
	0.5	120	7.8	28	1	0.8	− 5.8	1.2
	1	120	7.8	28	4	0.6	− 6.9	0.6
	2	120	7.8	40	3	0.6	− 6.8	1.8
	5	120	7.8	63	6	0.6	− 5.1	0.9
	10	120	7.9	36	1	0.5	− 9.2	0.5
Ag Sigma 576832	0.2	24	7.8	26	1	0.7	− 7.9	1.3
	0.5	24	7.8	43	13	0.4	− 9.5	0.7
	1	24	7.8	138	11	0.2	− 10.9	1.6
	2	24	7.8	244	166	0.3	− 10.0	0.8
	5	24	7.8	273	186	0.4	− 10.4	1.3
	10	24	7.8	308	36	0.3	− 11.3	0.8
	0.2	120	8.1	79	2	0.4	− 11.1	1.6
	0.5	120	8.0	73	2	0.4	− 11.2	0.3
	1	120	7.9	117	3	0.6	− 7.7	0.8
	2	120	8.1	89	3	0.4	− 10.8	0.9
	5	120	7.9	109	16	0.4	− 9.3	0.8
	10	120	7.9	150	16	0.3	− 7.5	0.7
BaSO_4_ NM 220	0.2	24	7.6	20	1	0.4	− 8.6	0.5
	0.5	24	7.6	27	2	0.7	− 9.5	0.8
	1	24	7.6	21	1	0.5	− 8.1	0.9
	2	24	7.6	21	1	0.6	− 9.6	0.9
	5	24	7.7	32	1	0.8	− 9.6	0.2
	10	24	7.7	41	3	1.0	− 11.6	0.8
	0.2	120	7.8	51	1	0.6	− 4.1	0.9
	0.5	120	7.8	54	2	0.6	− 7.7	0.4
	1	120	7.8	59	1	0.6	− 8.1	0.7
	2	120	7.8	66	2	0.6	− 4.6	1.3
	5	120	7.8	80	1	0.6	− 8.3	1.0
	10	120	7.8	95	1	0.6	− 10.0	0.8
CeO_2_ NM 212	0.2	24	7.6	21	2	0.5	− 6.5	0.4
	0.5	24	7.6	24	1	0.7	− 7.4	0.8
	1	24	7.7	109	53	0.4	− 6.6	0.2
	2	24	7.7	351	218	0.4	− 10.2	0.6
	5	24	7.7	598	446	0.8	− 8.1	0.9
	10	24	7.7	392	116	0.5	− 10.7	1.0
	0.2	120	8.2	76	1	0.4	− 7.0	1.3
	0.5	120	7.9	56	3	0.6	− 7.3	0.4
	1	120	7.8	98	4	0.8	− 4.5	0.5
	2	120	7.8	90	42	0.7	− 6.7	0.3
	5	120	7.9	114	1	0.6	− 9.4	0.7
	10	120	7.9	194	29	0.4	− 9.1	0.6

Dissolution studies in the cell incubation media showed similar trends as the biological clearance data summarized by Arts et al., but there are apparent disagreements with the REACH grouping categories [32]. ZnO showed high 24 h solubility (~10.0 µg/mL) followed by BaSO_4_ (2.4 µg/mL) and Ag (0.01 µg/mL), while dissolution of TiO_2_ and CeO_2_ was not observed (Table 1) [32]. This information suggests that the applied realistic doses for ZnO are borderline to the 24 h solubility level in the test media. As a result, the associated detrimental effects to the HepG2 cells in the experiments with ZnO may, to a great extent, be induced by the dissolved Zn^2+^ ions and not the ENM. With regards to CeO_2_, it was observed that 2.4 µg/mL was dissolved after 24 h. Consequently, particle-induced effects were not expected until the second-highest dose applied in this study, unless the effects are very acute.

**Fig. 1:**
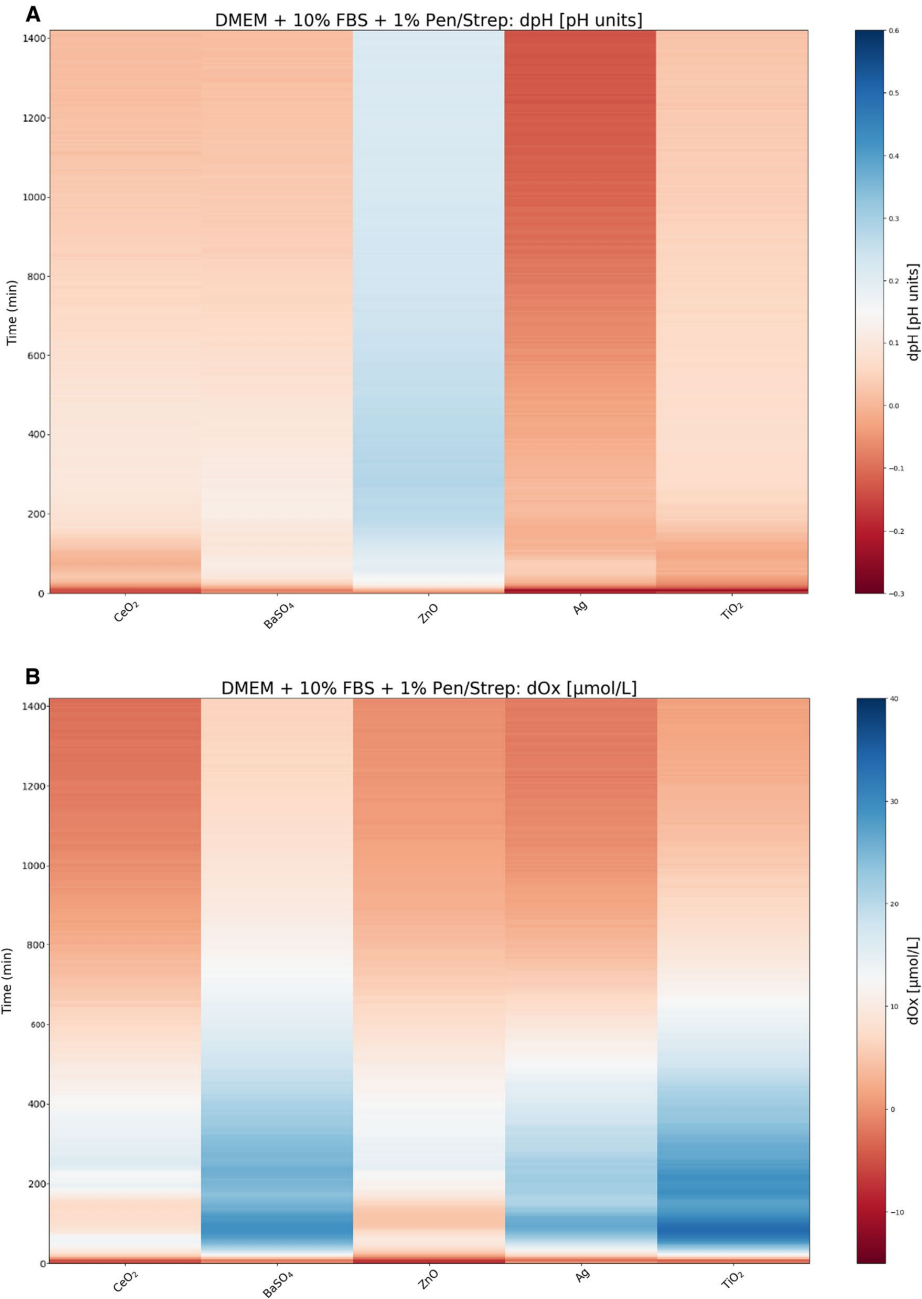
Surface plot shows the 24 h (1400 min) temporal pH reactivity (dpH) (**A**) and temporal oxidative reactivity (dOx) (**B**) for CeO_2_ (NM-212), BaSO_4_ (NM-220), ZnO (NM-111), Ag (Sigma 576832) and TiO_2_ (NM-105) in DMEM + 10% FBS + 1% Pen/Strep cell culture medium, during in vitro test conditions using the SDR method

Analysis of the particle pH and oxygen (O_2_) reactivities (Fig. 1) in the test medium showed minor effect with an increase in pH (~0.2–0.3 pH units) for ZnO. This is a lower pH effect than reported in Da Silva et al., where pH increased to above pH 9 in Hams F12 + 10% FBS + 1% Pen/Strep [36, 37]. Changes in the pH of the test medium are driven by the dissolution of the ENMs into different ions. For example, the observed pH increase for ZnO, which mainly occurred within the first 15 mins of the test, is explained by the dissolution of ZnO into Zn^2+^ ions and two hydroxide ions. Similar fast kinetics was observed by the pH increase in this study too. In contrast to ZnO, all the other 4 materials resulted in a pH decrease. For BaSO_4_, the decrease was minor, while it was pronounced and persistent for Ag (~ −0.2 pH units).

The temporal oxidative (dO_2_) reactivity is understood directly as the extent by which the test material, as a result of redox reactivities and potential dissolution, causes changes in the O_2_ concentration dissolved in the test medium with and without the presence of the ENM. With regards to the oxidative behaviour (Fig. 1B), BaSO_4_, Ag, and TiO_2_ showed a moderate increase in O_2_ within the first 500–700 mins after which the oxidative reaction is neutralised. On a relative scale between the 5 test materials, TiO_2_ followed by BaSO_4_ appear to be the materials with the highest initial dO_2_ reactivity. The relatively short duration of the observed reactivity suggests that the potential biological effect of material-induced changes in dO_2_ will be due to reactions within the first 200–600 mins after exposure is initiated.

### Single bolus, acute and prolonged ENM exposures

Acute, single bolus ENM exposures are commonly used for in vitro ENM hazard assessment and were undertaken to establish a foundation for which to compare prolonged and repeated ENM exposure regimes against.

#### Liver functionality: albumin and urea

To establish that no significant loss to liver functionality in the 3D HepG2 models occurred and that their fidelity was maintained following either acute (24 h) or prolonged (120 h) ENM exposures, the levels of both albumin and urea were assessed.

Albumin levels were found to remain relatively stable across the dose-range for each test ENM evaluated (Table 3). The concentration of albumin was generally greater following longer-term culture than acute exposure periods, which is to be expected as albumin accumulates with increasing culture time of the 3D spheroids [29]. There was no significant change in albumin levels following longer-term exposure to any of the ENMs tested, nor with acute exposure to TiO_2_, ZnO or CeO_2_. However, a significant *(p* ≤ *0.05)* reduction in albumin was observed following 24 h exposure to the higher doses (5.0 µg/mL and 10.0µg/mL) of Ag and BaSO_4_ ENMs.

**Table 3:** Albumin concentration per HepG2 spheroid following both acute (24 h) and prolonged (120 h) ENM exposure to increasing concentrations (0.2–10.0 μg/mL) of ENMs

3D HepG2 liver spheroid liver functionality	Albumin per spheroid (ng/µL) following Acute (24 h) exposure (95% CI)					Albumin per spheroid (ng/µL) following Prolonged (120 h) exposure (95% CI)				
ENM concentration (µg/mL)	TiO_2_	ZnO	Ag	BaSO_4_	CeO_2_	TiO_2_	ZnO	Ag	BaSO_4_	CeO_2_
Untreated negative control	**29.660 **(4.85–24.47)	**23.171 **(15.14–30.20)	**27.785 **(10.61–44.96)	**30.703 **(15.06–46.35)	**52.878 **(42.54–63.22)	**35.783 **(31.02–40.54)	**27.510 **(21.82–33.20)	**36.944 **(25.88–38.01)	**32.550 **(16.19–48.91)	**49.851 **(41.15–58.56)
0.2	**28.235 **(23.88–32.59)	**24.715 **(19.06–30.37)	**24.903 **(19.12–30.69)	**27.002 **(21.50–32.51)	**52.235 **(45.47–59.00)	**35.245 **(26.64–43.85)	**28.506 **(21.66–35.35)	**33.821 **(23.58–44.06)	**31.043 **(25.36–36.72)	**50.506 **(45.50–55.52)
0.5	**28.622 **(22.52–34.72)	**24.232 **(17.51–30.95)	**33.376 **(5.90–60.86)	**26.915 **(20.44–33.39)	**55.154 **(47.86–62.45)	**37.734 **(34.45–41.02)	**26.990 **(21.17–32.81)	**45.420 **(37.39–53.45)	**32.922 **(29.74–36.11)	**49.160 **(35.30–63.02)
1.0	**29.108 **(26.00–32.22)	**23.943 **(16.90–30.99)	**24.207 **(16.36–32.06)	**25.417 **(16.71–34.12)	**56.472 **(41.31–71.63)	**34.880 **(24.30–45.46)	**27.748 **(21.07–34.42)	**30.996 **(25.10–36.90)	**35.691 **(31.84–39.54)	**52.407 **(51.08–53.74)
2.0	**32.104 **(24.97–39.24)	**24.343 **(14.05–34.64)	**33.428 **(19.10–47.76)	**29.790 **(17.75–41.83)	**52.620 **(47.75–57.49)	**33.071 **(26.30–39.85)	**27.132 **(23.13–31.13)	**40.737 **(35.84–45.63)	**38.078 **(22.41–53.57)	**50.857 **(43.77–57.94)
5.0	**30.114 **(16.48–43.75)	**24.611 **(21.88–27.34)	9.520* (− 7.69 to 26.74)	18.216* (6.51–29.92)	**55.937 **(42.99–68.88)	**33.236 **(28.45–38.02)	**28.740 **(23.08–34.40)	**35.984 **(25.21–46.76)	**30.937 **(29.41–32.46)	**56.619 **(45.51–67.73)
10.0	**28.137 **(21.96–34.32)	**22.989 **(14.95–31.03)	12.666* (−5.14 to 30.47)	14.032* (4.27–23.79)	**51.445 **(43.73–59.16)	**30.494 **(24.01–36.97)	**31.111 **(15.22–47.01)	**35.342 **(32.29–38.39)	**33.686 **(30.99–36.39)	**50.287 **(46.88–53.70)
Aflatoxin B1 positive control	**24.488 **(4.24–44.74)	**22.925 **(20.29–25.56)	**32.943 **(5.80–60.08)	**35.329 **(10.87–59.79)	**52.907 **(47.02–58.80)	**36.650 **(31.60–41.70)	**30.789 **(13.38–49.20)	**38.405 **(33.32–43.49)	**31.139 **(30.14–32.14)	**48.498 **(44.01–52.99)

Mean data of three biological replicates, analysed in triplicate (n = 9) are presented with 95% confidence intervals. Significance is indicated in relation to the negative control, where * = *p* ≤ *0.05*

In a similar manner to albumin, the concentration of urea produced by the HepG2 spheroids also remained consistent across all ENM exposures (Table 4). A significant reduction *(p* = *0.0061)* in urea was only observed following acute exposure to 10.0 µg/mL of ZnO and longer-term exposure to 5.0 µg/mL of TiO_2_.

**Table 4:** Urea concentration per HepG2 spheroid following both acute (24 h) and prolonged (120 h) exposure to increasing concentrations (0.2–10.0 µg/mL) of ENMs

3D HepG2 liver spheroid liver functionality	Urea per spheroid (ng/µL) following Acute (24 h) exposure (95% CI)					Urea per spheroid (ng/µL) following Prolonged (120 h) exposure (95% CI)				
ENM concentration (µg/mL)	TiO_2_	ZnO	Ag	BaSO_4_	CeO_2_	TiO_2_	ZnO	Ag	BaSO_4_	CeO_2_
Untreated negative control	**0.690 **(0.625–0.754)	**0.937 **(0.786–1.088)	**0.455 **(0.217–0.693)	**0.394 **(0.235–0.553)	**0.675 **(0.564–0.785)	**1.021 **(0.981–1.061)	**0.744 **(0.365–1.123)	**0.389 **(0.188–0.589)	**0.292 **(0.126–0.459)	**0.927 **(0.746–1.107)
0.2	**0.813 **(0.676–0.950)	**1.245 **(1.097–1.393)	**0.520 **(0.337–0.703)	**0.498 **(0.379–0.618)	**0.724 **(0.697–0.752)	**1.108 **(0.902–1.314)	**0.832 **(0.565–1.098)	**0.523 **(0.431–0.615)	**0.402 **(0.276–0.527)	**0.968 **(0.812–1.124)
0.5	**0.889 **(0.809–0.970)	**1.036 **(0.969–1.103)	**0.550 **(0.307–0.793)	**0.505 **(0.312–0.698)	**0.762 **(0.649–0.874)	**1.273 **(0.811–1.725)	**0.769 **(0.563–0.974)	**0.563 **(0.440–0.686)	**0.409 **(0.207–0.611)	**0.920 **(0.761–1.078)
1.0	**0.859 **(0.693–1.025)	**1.145 **(1.056–1.234)	**0.539 **(0.414–0.664)	**0.638 **(0.571–0.706)	**0.775 **(0.735–0.816)	**1.213 **(1.092–1**.**334)	**0.794 **(0.675–0.914)	**0.490 **(0.264–0.716)	**0.549 **(0.478–0.621)	**0.940 **(0.710–1.170)
2.0	**0.930 **(0.783–1.076)	**1.120 **(0.873–1.366)	**0.567 **(0.423–0.771)	**0.573 **(0.376–0.771)	**0.717 **(0.549–0.885)	**1.197 **(0.902–1.492)	**0.795 ** (0.502–1.089)	**0.521 **(0.428–0.615)	**0.481 **(0.274–0.688)	**0.971 **(0.706–1.236)
5.0	**0.699 **(0.565–0.834)	**0.793 **(0.668–0.919)	**0.596 **(0.324–0.869)	**0.643 **(0.487–0.799)	**0.782 **(0.645–0.919)	0.805* (0.636–0.974)	**0.832 **(0.729–0.935)	**0.703 **(0.595–0.811)	**0.781 **(0.515–1.047)	**0.955 **(0.672–1.238)
10.0	**0.628 **(0.371–0.885)	0.728* (0.635–0.821)	**0.571 **(0.518–0.624)	**0.618 **(0.214–1.021)	**0.792 **(0.617–0.967)	**0.846 **(0.771–0.921)	**0.737 **(0.532–0.942)	**0.726 **(0.367–1.085)	**0.899 **(0.735–1.063)	**0.909 **(0.755–1.064)
Aflatoxin B1 Positive Control	**0.773 **(0.718–0.828)	**0.881 **(0.729–1.033)	**0.515 **(0.332–0.698)	**0.423 **(0.330–0.517)	**0.755 **(0.500–1.010)	**0.846 **(0.771–0.921)	0.488* (0.212–0.763)	**0.516 **(0.432–0.600)	**0.323 **(0.224–0.422)	**0.953 **(0.878–1.029)

Mean data of three biological replicates, analysed in triplicate (n = 9) are presented with 95% confidence intervals. Significance is indicated in relation to the negative control, where * = *p* ≤ *0.05*

#### (Pro)-inflammatory response: IL-8, IL-6 and TNF-α cytokine release

**Fig. 2:**
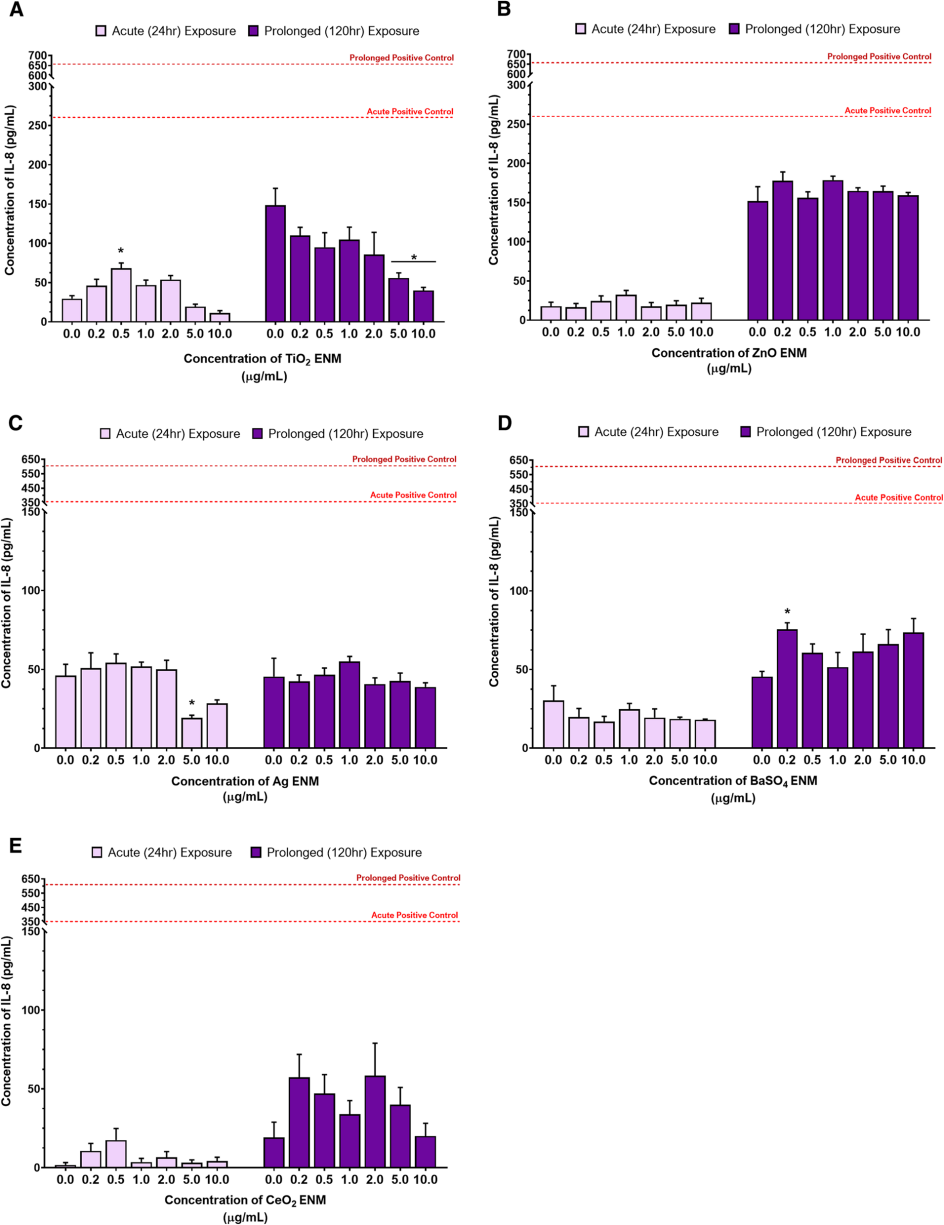
Release of IL-8 (pro-)inflammatory cytokines in 3D HepG2 liver spheroids following both acute (24 h) and longer-term (120 h) exposure to 0.2–10.0 µg/mL of (**A**) TiO_2_, (**B**) ZnO, (**C**) Ag, (**D**) BaSO_4_ and (**E**) CeO_2_ ENMs. An untreated, media only sample was used as the negative control. The positive assay control was 0.25 µg/mL of TNF-α protein (NBP2-35076-50 µg, Biotechne, UK), as indicated by the dotted line, which represents the mean positive control response for both acute (light red line) and prolonged (dark red line) exposures. Mean data of three biological replicates, analysed in triplicate (n = 9) are presented ± SEM. Significance is indicated in relation to the negative control, where * = *p* ≤ *0.05*

Following ENM exposure, IL-6, IL-8 and TNF-α cytokine levels were assessed to investigate the induction of any potential (pro-)inflammatory response. Attributable to the 3D liver spheroid model being a monoculture of HepG2 epithelial-like cells, all IL-6 and TNF-a results were found to be below detectable limits regardless of ENM tested or exposure-regime applied and so these cytokines were not considered further (data not shown). In contrast, IL-8, an acute phase chemokine released by hepatic epithelial cells, was modified in response to the ENM exposures as illustrated in Fig. 2. When comparing the IL-8 response between acute and prolonged exposure regimes for TiO_2_, ZnO, BaSO_4_ and CeO_2_, there was an increase in the concentration of IL-8 present across the dose range. Exposure to 0.5 µg/mL of TiO_2_ induced the only significant *(p* = *0.0042)* increase in IL-8 following acute exposure, which was no longer observed after 120 h (Fig. 1A). Instead, as the concentration of TiO_2_ increased in the longer-term exposure, the concentration of IL-8 present decreased with 5.0 µg/mL and 10.0 µg/mL TiO_2_ inducing a significant (*p* ≤ *0.01*) reduction in IL-8. Neither ZnO (Fig. 2B), or Ag (Fig. 2C) induced any significant changes in IL-8 production following either acute or prolonged low-dose ENM exposure with both showing a similar trend to the control across the dose range. Fig. 2D demonstrates that BaSO_4_ was the only material to induce an increase in IL-8 across the 2.0–10.0 µg/mL dose range. However, significance *(p* = *0.0261)* was only achieved at the single dose of 0.2 µg/mL BaSO_4_. Exposure to CeO_2_, Fig. 2E, resulted in IL-8 induction at 0.2 µg/mL and 0.5 µg/mL following acute exposures and across the prolonged exposure dose range (0.2 and 2.0 µg/mL). However, none of these IL-8 peaks were found to be significant despite being up to 3-fold higher than the negative control.

#### Cytotoxicity and genotoxicity

To determine if the test materials induced fixed DNA damage following both acute and prolonged exposure, the micronucleus (MN) assay was employed in conjunction with an appropriate cytotoxicity assay.

**Fig. 3:**
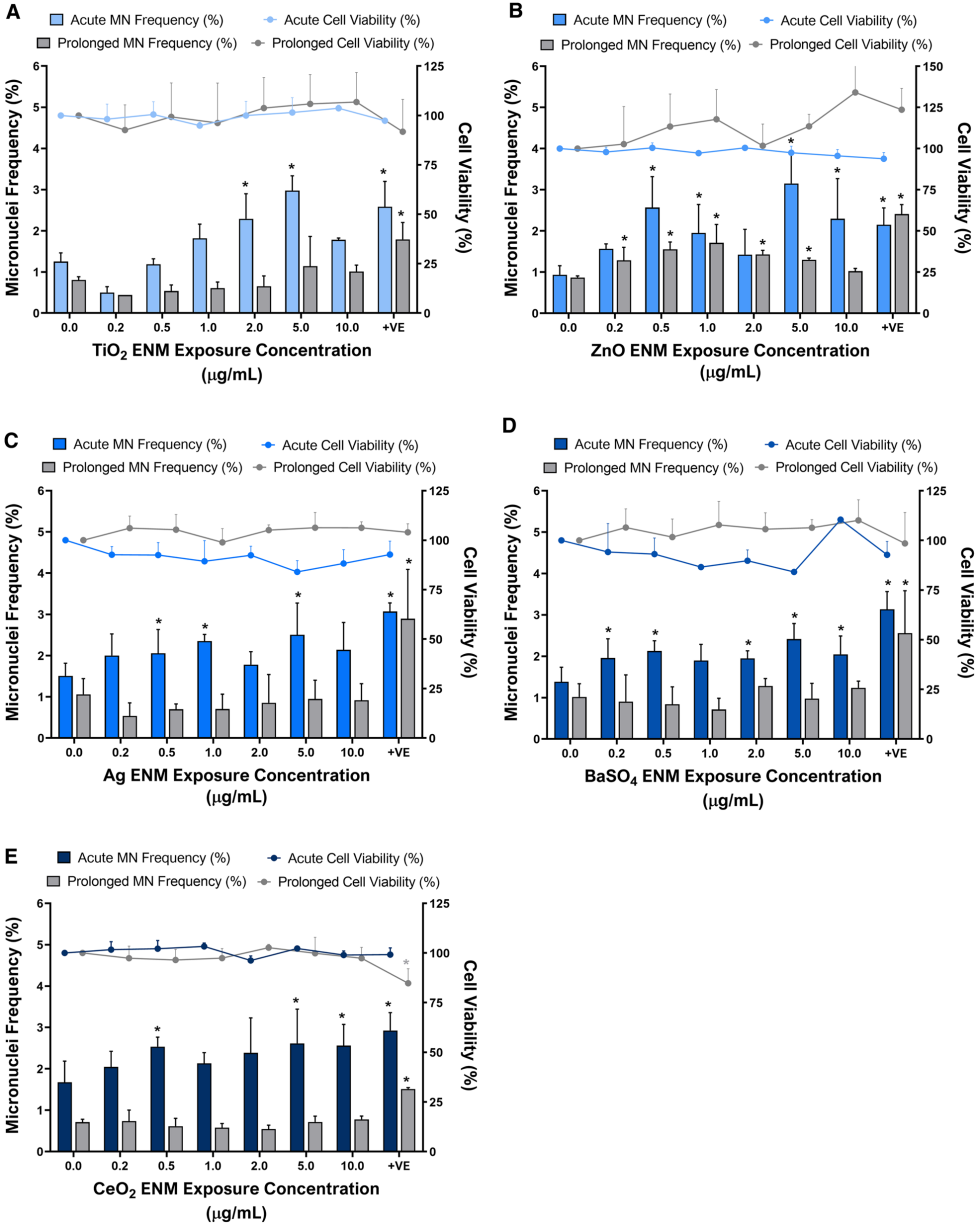
Cytotoxicity and genotoxicity responses in HepG2 spheroids following both acute (24 h) and prolonged (120 h) exposure to 0.2–10.0 µg/mL of (**A**) TiO_2_, (**B**) ZnO, (**C**) Ag, (**D**) BaSO_4_ and (**E**) CeO_2_ ENMs. Cytotoxicity was assessed using the cytokinesis-block proliferation index (CBPI) for acute exposures, whilst trypan blue was assessed for the prolonged exposures, both of which are presented relative to the negative, untreated control. A known liver carcinogen, aflatoxin B1 (0.1 µM) was used as a positive control for genotoxicity. For acute exposures, 1000 binucleated cells were scored per replicate for each dose point using the cytokinesis-block version of the MN assay (2000 binucleate cells scored in total per dose). For prolonged exposures, 2000 mononucleated cells were scored per replicate for each dose point using the mononuclear MN assay (4000 mononucleate cells scored in total per dose). Mean data of two and three biological replicates (n = 2, n = 3) for genotoxicity and cytotoxicity respectively is presented ± SD. Significance indicated in relation to the negative control: * = *p* ≤ *0.05*

As shown in Fig. 3, cytotoxicity was not induced following either acute or prolonged exposure to any of the test ENM up to a top dose of 10.0 µg/mL of material. In contrast, a significant dose-dependent increase in genotoxicity was observed with all test ENMs following acute exposure. With TiO_2,_ the lowest observed adverse effect level (LOAEL) was 2.0 µg/mL (*p* = *0.0052*); the frequency of MN induction increased further at 5.0 µg/mL (*p* < *0.0001*), where the MN frequency was 2.4-fold higher than the negative control. Similar to TiO_2_, whilst Ag, BaSO_4_ and CeO_2_ did not induce a significant increase in genotoxicity following prolonged exposures, each material was shown to induce a significant acute genotoxic response. Ag (Fig. 3C) displayed a significant increase in genotoxicity following 24 h exposure to 0.5 µg/mL *(p* = *0.0033)*, 1.0 µg/mL *(p*=*0.0004)* and 5.0 µg/mL *(p* = *0.0032);* although the observed effect was a plateau with all doses exhibiting an MN frequency 2.05–2.51%. BaSO_4_ induced a significant genotoxic response with all but one concentration, 1.0 µg/mL, following acute exposure as illustrated in Fig. 3D. Similarly to Ag, acute exposure to CeO_2_ (Fig. 3E) induced a significant genotoxicity response at 3 concentrations: 0.5 µg/mL *(p* = *0.0185)*, 5.0 µg/mL *(p *= *0.0191)* and 10.0 µg/mL *(p* = *0.0209)*. ZnO (Fig. 3B) was the only material to exhibit both an acute and prolonged effect upon genotoxicity in 3D HepG2 liver spheroids. ZnO appears to induce different patterns of genotoxicity between the acute and prolonged exposure regimes. The acute genotoxic response appears to peak and trough, whereby ZnO induces a significant increase in genotoxicity at 0.5 µg/mL (*p* < *0.0001*), 1.0 µg/mL (*p* = *0.0083*), 5.0 µg/mL (*p* < *0.0001*) and 10.0 µg/mL *(p* = *0.0001*). In contrast, following the prolonged exposure, genotoxicity increased in a dose dependent manner up to 1.0 µg/mL and then plateaued, with the top dose reducing back to control levels. As shown by Fig. 3, none of the other test materials induced a significant positive induction of genotoxicity following prolonged exposure. Considering the acute (24 h) data, the genotoxicity potency ranking based on the dose response relationship and the greatest fold-change in MN induction is: ZnO > TiO_2_ > BaSO_4_ = CeO_2_ > Ag.

### Fractionated repeated, prolonged ENM exposures

**Fig. 4:**
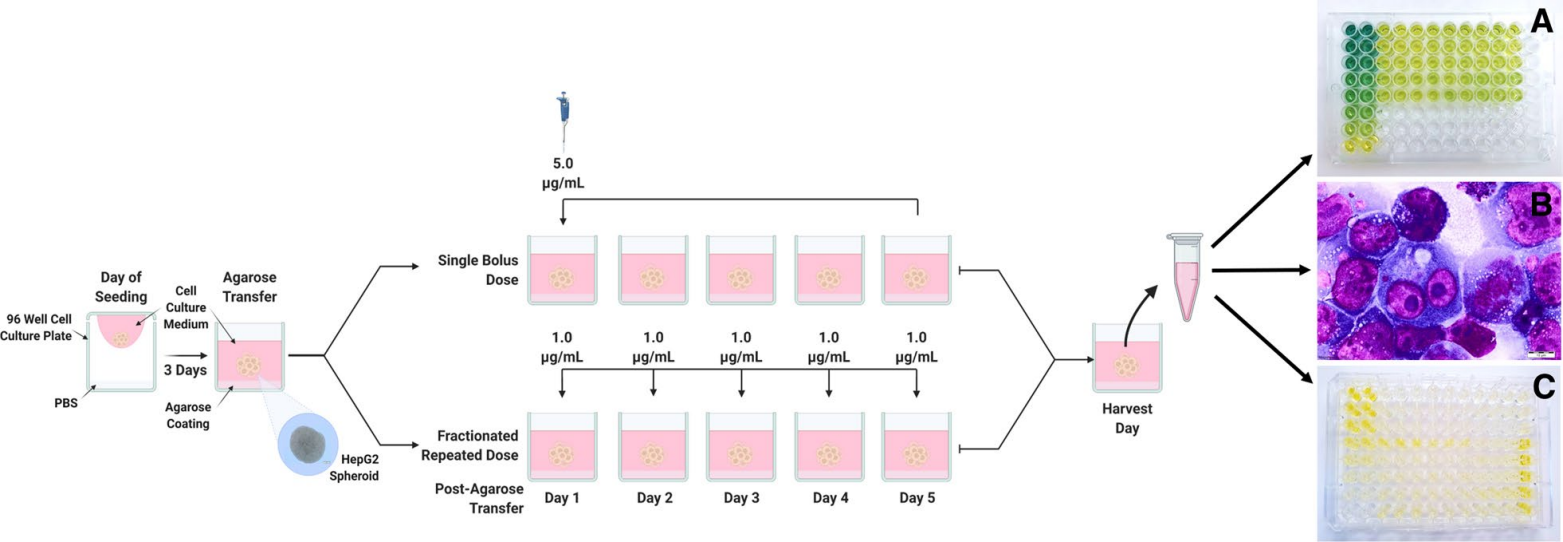
Schematic representation of the prolonged (120 h) single, bolus ENM exposure regime and the fractionated, repeated ENM exposure regime assessed using 3D HepG2 spheroids and key biochemical endpoint analysis techniques selected. **A** Colourmetric based assay for albumin quantification. **B** CellSens X63 image displaying binucleate formation following the cytokinesis-block micronucleus (CBMN) assay with the presence of a micronucleus. **C** (Pro)-inflammatory ELISAs for Interleukin 8 (IL-8), Interleukin-6 (IL-6) and Tumor Necrosis Factor Alpha (TNF-α). Created with Biorender.com

Fractionated, repeated prolonged ENM exposures were also investigated to more accurately simulate human ENM exposure and to determine if the added complexity of the exposure regime would significantly affect the toxicological outcome in 3D HepG2 liver spheroids. Whilst both exposure regimes resulted in the same final exposure concentration, the manner in which the ENMs were exposed to the spheroids differed between a single, bolus dose on day one and a repeated, fractionated dose given every day for the entire five day exposure, as illustrated in Fig. 4.

#### Liver functionality: albumin and urea

To ensure the HepG2 spheroids maintained their phenotypic functionality following both ENM prolonged exposure regimes, the levels of both albumin and urea were measured post exposure. For both albumin and urea concentrations, there was no significant difference between prolonged single, bolus, and repeated, fractionated exposure regimes irrespective of the test ENM applied or the dose. Based on the values in Table 5, the average range between the mean albumin and urea values for each exposure regime was 3.84 ng/µL of albumin and 0.06 ng/µL of urea. Thus, the manner in which the 3D HepG2 liver spheroids are exposed to ENM over prolonged exposure regimes was not observed to significantly impede liver functionality.

**Table 5:** Concentration of albumin and urea produced per HepG2 spheroid following a prolonged (120 h) exposure using a single, bolus dosing regime or a fractionated, repeated dosing regime with 0.5 and 5.0 µg/mL of TiO_2_ and ZnO ENMs

3D HepG2 liver spheroid liver functionality	Albumin per spheroid (ng/µL) (95% CI)		Urea per spheroid (ng/µL) (95% CI)	
Dosing regime employed	Bolus	Fractionated	Bolus	Fractionated
Untreated negative control	**32.807 **(29.323–36.291)		**0.805 **(0.729–0.880)	
TiO_2_ 0.5 µg/mL	**37.736 **(29.973–45.499)	**33.716 **(30.426–37.007)	**0.885 **(0.722–1.048)	**0.806 **(0.616–0.995)
TiO_2_ 5.0 µg/mL	**32.740 **(29.457–36.022)	**31.289 **(25.383–37.195)	**0.818 **(0.757–0.878)	**0.815 **(0.772–0.858)
ZnO 0.5 µg/mL	**42.960 **(30.320–55.601)	**32.948 **(28.644–37.252)	**0.790 **(0.585–0.996)	**0.819 **(0.781–0.858)
ZnO 5.0 µg/mL	**33.971 **(31.280–36.662)	**36.665 **(33.365–39.965)	**0.854 **(0.775–0.932)	**0.666 **(0.492–0.840)
Aflatoxin B1 positive control	**35.969 (**25.594–46.344)	**36.978 **(30.590–43.366)	**0.769 **(0.707–0.885)	**0.792 **(0.758–0.826)

Mean data of three biological replicates, analysed in triplicate (n = 9) are presented with 95% confidence intervals. Significance is indicated in relation to the negative control, where * = *p* ≤ *0.05*

#### (Pro)-inflammatory response: IL-8, IL-6 and TNF-α cytokine release

**Fig. 5:**
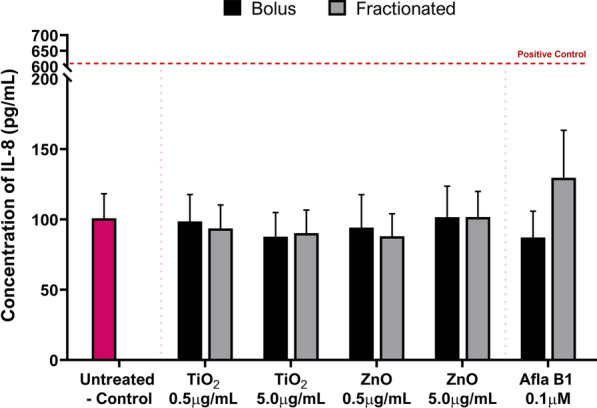
Release of IL-8 (pro-)inflammatory cytokines in 3D HepG2 liver spheroids following a prolonged (120 h) exposure using a single, bolus dosing regimen or a fractionated, repeated dosing regimen with 0.5 and 5.0 µg/mL of TiO_2_ and ZnO ENMs. An untreated, media only sample was used as the negative control whilst 0.25 µg/mL of TNF-α protein (NBP2-35,076-50ug, Biotechne, UK) was used as the positive assay control, as indicated by the red dotted line which represents the mean positive control response. Mean data of three biological replicates, analysed in triplicate (n = 9) are presented ± SEM. Significance indicated in relation to the negative control: * = *p* ≤ *0.05*

With the complex interplay of inflammatory mediators, feedback loops and pathway cascades, timing is crucial with inducing a (pro-)inflammatory response. Therefore, it was important to establish if modifying the prolonged exposure regime to a repeated, fractionated exposure method as opposed to a single, bolus exposure on day one, would affect the (pro-)inflammatory response in HepG2 spheroids. In a similar manner to the acute and prolonged exposure studies described earlier, IL-6, IL-8 and TNF-α cytokine release was assessed for both the prolonged single, bolus and the repeated, fractionated ENM exposure regimes, but only an IL-8 (pro-)inflammatory release was detectable (Fig. 5). Prolonged exposure to both 0.5 µg/mL and 5.0 µg/mL of TiO_2_ and ZnO ENMs dosed via the two different methods, showed no significant difference in the IL-8 (pro-)inflammatory response in the HepG2 liver spheroids. For the individual test ENMs, there appears to be little to no difference at all in the concentration of IL-8 released following exposure to either material.

#### Cytotoxicity and genotoxicity

**Fig. 6:**
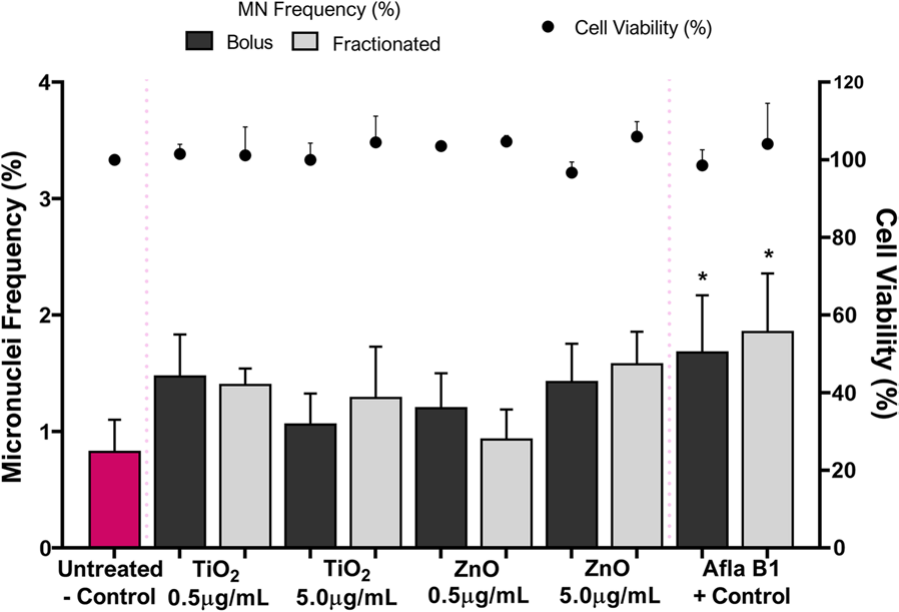
Cytotoxicity and genotoxicity responses in HepG2 spheroids following a prolonged (120 h) exposure using a single, bolus dosing regimen or a fractionated, repeated dosing regimen with 0.5 and 5.0 µg/mL of TiO_2_ and ZnO ENMs. Cytotoxicity was assessed using the trypan blue exclusion assay and is presented relative to the negative, untreated control. A known liver carcinogen, aflatoxin B1 (0.1 µM) was used as a positive control for genotoxicity. For acute exposures, 1000 binucleated cells were scored per replicate for each dose point using the cytokinesis-block version of the MN assay (2000 binucleate cells scored in total per dose). For prolonged exposures, 2000 mononucleated cells were scored per replicate for each dose point using the mononuclear MN assay (4000 mononucleate cells scored in total per dose). Mean data of two and three biological replicates (n = 2, n = 3) for genotoxicity and cytotoxicity respectively is presented ± SD. Significance indicated in relation to the negative control: * = *p* ≤ *0.05*

To mimic a gradual accumulation of ENMs and determine the effect this may have on DNA damage and cytotoxicity in HepG2 liver spheroids, spheroids were dosed with TiO_2_ and ZnO ENMs via two techniques; a single, bolus dose or a repeated, fractionated dose. Exposing 3D HepG2 liver spheroids to either TiO_2_ or ZnO, irrespective of dose, did not induce a significant increase in cell death or MN frequency as compared to the untreated control (Fig. 6). Furthermore, there was no significant difference in the cytotoxicity or genotoxicity observed when comparing the single, bolus dose on day one versus the repeated, fractionated dose every day in 3D HepG2 liver spheroids.

## Discussion

It is widely acknowledged that it is unsustainable and ethically divisive to rely primarily on in vivo based test systems for comprehensive ENM hazard assessment, as such, research into suitable, physiologically relevant in vitro alternatives has been at the forefront of nanotoxicology in recent years. Not only have scientists strived to alleviate the limitations of current in vitro models to enhance the longevity and predictivity of the models, but, as in the present study, there have been recent efforts to start addressing the manner in which humans are exposed to ENMs in natural life [38, 39]. This study aimed to determine, firstly, if a more realistic, low-dose prolonged ENM exposure would provoke a significantly different (geno)toxicological effect compared to an acute exposure in 3D HepG2 spheroids. Secondly, this study aimed to determine if a daily repeated, fractionated ENM exposure regime would significantly alter the toxicological outcome in 3D HepG2 spheroids compared to a single, bolus prolonged exposure. To assess this, a cell-line based 3D in vitro HepG2 spheroid model able to evaluate cytotoxicity, (pro-)inflammatory response and genotoxicity associated with both acute and longer-term (≤ 10 days) ENM exposure upon the liver was utilised. In this system, hepatocyte spheroids recapitulated basic in vivo hepatic functions and structure, whilst maintaining specific parameters required for multiple biochemical endpoint testing [28, 29]. Applying this liver model, a range of five different ENMs were evaluated, across a low concentration range of 0.2–10.0 µg/mL, to determine whether the individual physico-chemical characteristics would elicit a different biological response following either an acute (24 h) or longer-term (120 h) exposure scenario in vitro. A low-dose concentration range was selected to not only simulate physiological relevant concentrations of translated ENMs in the liver, but to ensure that the in vitro test system is not over saturated by the sheer volume of material, which increases the risk of a misleading positive toxicity result [14, 15].

Previously, many in vitro studies have focused on the acute effects of high concentrations of ENMs [17, 40]. Acute exposure regimes are a less laborious and generally a more efficient way to quickly determine whether a substance has the potential to illicit an adverse reaction or be hazardous. However, it does not provide an accurate representation of the prolonged effects this acute exposure may have nor does it provide any indication of the accumulated effects were this exposure to be a recurring event. In order to address this, a longer-term exposure regime of five days (120 h; bolus and repeated) was established to provide a more realistic dosing scenario, as most individuals are likely to be exposed to multiple, low doses of ENMs over time [17, 41]. Alongside the evaluation of key toxicological endpoints, such as cytotoxicity, (pro-)inflammatory response and genotoxicity, the viability and fidelity of the liver model had to be assessed throughout the duration of this study. As biomarkers of hepatic metabolism and functionality, albumin and urea production were measured. Albumin is a stable, 66.5 kDa plasma protein primarily synthesised in the liver and is principally responsible for maintaining oncotic pressure within in the blood, in order to prevent excess volumes of water being leaked into the surrounding tissues [42]. In addition to this, albumin has been found to play a role in immunomodulation, antioxidant effects and binding to multiple drugs, toxins, and other molecules, including ENMs. Albumin is one of the most abundant proteins frequently found in the protein corona of ENMs [43, 44]. Urea is an organic, 60 Da, metabolic end product of protein catabolism; a process which happens within the liver as it is the sole organ that has enzymes for urea synthesis [45]. Urea synthesis is crucial in the breakdown and excretion of nitrogen waste products, such as ammonia, which are toxic to the mammalian body if not metabolised to urea and excreted as urine [46]. Across all acute ENM exposures, the viability and liver functionality was not significantly reduced, with the exception of exposure to the top concentrations (5.0 µg/mL and 10.0 µg/mL) of Ag and BaSO_4_ which did significantly reduce albumin production. As this reduction was not mirrored in the production of urea, one suggestion for this decrease could be the tendency of ENMs to actively adsorb proteins, like albumin, to their surface as part of the protein corona; the reduction may therefore be an artifact [47, 48]. Over the duration of the prolonged exposures, as expected, the albumin levels increased as a result of the actively proliferating cells on the outer layers of the spheroid. Subsequently, the higher prevalence of albumin could saturate the ENM corona and so the previously observed decrease in albumin may have been compensated for. Overall, neither acute nor prolonged exposure to these test ENMs significantly reduced the fidelity of these HepG2 spheroids, which also correlates with the limited cytotoxicity observed over the concentration-ranges and exposure regimes applied.

Each ENM has a unique set of physico-chemical characteristics (e.g*.* size, shape, composition, surface charge, coating, crystallinity and solubility) which determine how these materials interact with biological systems; influencing cellular uptake, bio-durability, translocation and deposition around the body [49]. Not only is it important to fully characterize an ENM prior to exposure, it is equally as important to characterize these materials under biological exposure conditions as these materials may undergo transformation (e.g. dissolution, aggregation and reprecipitation) when they come into contact with different biological fluids [39]. As a result, these novel size-specific characteristics often heavily influence the toxicological potential for such materials. Therefore, it is particularly important to consider the physico-chemical characteristics and biotransformation potential of these materials when evaluating the toxicity outcomes observed.

Many ENMs are known reactive oxygen species (ROS)/reactive nitrogen species (RNS) inducers, and directly or indirectly cause an imbalance in the redox homeostasis of the cell [50–52]. Most metal based ENMs, particularly transition metals, elicit ROS and free radical mediated toxicity via Fenton-type reactions. As a result, their ability to influence intracellular calcium concentrations, activate (pro‐)inflammatory transcription factors (e.g., nuclear factor kappa B [NF‐kB]) and modulate cytokine release via the production of free radicals, is believed to be linked to the greater surface area, therefore increased surface reactivity, as well as the addition of pro-oxidant thiol groups on the surface of the materials [50–52]. Interestingly, the ENM with greatest surface area (TiO_2_) did exhibit the greatest O_2_ reactivity and induced the greatest IL-8 response over any other material, with a significant increase in IL-8 release observed following acute exposure to 0.50 µg/mL. This was no longer the case following prolonged exposures. Instead, as the concentration of TiO_2_ increased in the prolonged exposure studies, the concentration of IL-8 decreased. This may be attributable to the increased agglomeration observed, restricting cellular uptake and reducing the surface area available for oxidative reactions to occur. In addition, the likelihood is that any REDOX or Fenton-type reactions will have occurred within the first 24 h of the exposure. As a result, the production of free radicals that trigger the release of (pro-)inflammatory cytokines, like IL-8 via the activation of REDOX sensitive Nf-kβ or MAPK signalling pathways, may no longer be as actively expressed 120 h later. The differences in ENM associated IL-8 release between acute and prolonged exposures could suggest that other factors (e.g. dissolution, agglomeration, rate of cellular uptake) may be more influential in orchestrating the (pro-)inflammatory response during this time. It appears the two materials (Ag and ZnO) with the greatest solubility and lowest surface area, exhibit minimal dose-dependent effects in IL-8 release, with a consistent IL-8 response observed across almost all doses following either an acute or prolonged exposure. In contrast, ENMs with a more bio-persistent nature, which take longer to breakdown and clear, could cause a greater and more variable inflammatory response in the prolonged exposure. For example, BaSO_4,_ induced the only significant increase in IL-8 release following prolonged exposure. The overall increase in IL-8 release observed between the acute and prolonged ENM exposures is likely caused by the reduced oxygen diffusion towards the centre of the spheroid over time. This will result in increased hypoxic conditions within the spheroid core, which is associated with increased IL-8 production [29, 53–55].

Genotoxicity of the five test ENMs was assessed using the ‘gold standard’ in vitro MN assay, which is the recommended test for evaluating fixed gross chromosomal damage for regulatory purposes. Whilst there is an OECD Test Guideline (TG487) for this assay, it has long been recognised that nano-specific adaptations to the method are required, which were included in the approach taken within this study [56, 57]. No significant cytotoxicity was detected following either acute or prolonged exposure to any of the ENMs tested, regardless of the concentration or dosing regimen employed. However, all five ENMs tested positive for genotoxicity following acute exposure, albeit not in a dose-dependent manner, due to variation in agglomeration across dose ranges. A genotoxicity potency ranking was established based on the dose response and the greatest fold-change in MN induction, as follows: ZnO > TiO_2_ > BaSO_4_ = CeO_2_ > Ag. This genotoxicity potency ranking could help provide an insight into the DNA damaging potential of these ENMs if exposed to the human liver. However, it is also important to note that whilst for each material there is evidence of genotoxicity, there is also evidence of dose ranges where DNA damage does not occur and so there may be opportunity for safe exposure limits to be established. The average LOAEL post-acute exposure was induced by an ENM concentration of 0.5 µg/mL, with ZnO, BaSO_4_, CeO_2_ and Ag eliciting a 2.75-, 1.54-, 1.51- and 1.37-fold change in MN induction, respectively. For, TiO_2_, whilst the LOAEL was reached at 2.0 µg/mL with 1.83-fold increase in MN frequency over control, this material induced the second greatest increase in MN induction behind ZnO, with a 2.4-fold increase in MN fold following acute exposure to 5.0 µg/mL. The significant increase in MN induction following acute 24 h exposure to higher concentrations of TiO_2_, could be attributed to the high oxidative potential TiO_2_ exhibits within the first 24 h of exposure, Fig. 1B. This potent O_2_ reactivity suggests that the elevated DNA damage observed could be a result of ROS and oxidative stress. DNA is one of the major targets for oxidative stress induced damage (e.g. DNA–protein crosslinks, alkali-labile sites, DNA adducts, mutations), with OH^•^, a highly potent free radical, known to react with all components of DNA and causing strand breaks via the formation of 8-OHdG DNA adducts [58, 59]. Whilst TiO_2_ ENM O_2_ reactivity is very active during the first few hours of the exposure, this oxidative potential appears to decrease as exposure duration increases and so may no longer be as prominent following prolonged exposure, resulting in the lower MN frequency at 120 h. In all cases, the top dose of 10.0 µg/mL resulted in a lower MN frequency following acute exposure than that of the former dose of 5.0 µg/mL. This is most likely due to greater material agglomeration at the top dose, restricting ENM translocation through the compact spheroid structure, thus reducing cellular uptake and biological interaction. It is well known that at higher ENM concentrations, the degree of agglomeration tends to be greater than that at the lower concentrations of ENMs, as the high number of particles within a given space increases the chance of particle–particle interaction and subsequent agglomeration [60]. This was further illustrated in this study by the time- and dose-dependent increase in agglomeration, with the average agglomerate size increasing from 23 nm at 0.2 µg/mL to 282 nm at 10.0 µg/mL, and 37 nm at 0.2 µg/mL to 275 nm at 10.0 µg/mL, respectively (Table 2). Whilst, at the lowest concentration of 0.2 µg/mL, all five ENMs remain monodispersed following acute exposure and only small agglomerates (< 80 nm) had formed over the duration of the 120 h exposure. At lower doses, darkfield imaging of PHH microtissues following exposure to 1.25 µg/mL and 5.0 µg/mL of TiO_2_ (NM-105) and CeO_2_ (NM-212), illustrated that the ENMs could penetrate deep into the core, with a large proportion of the hepatocytes encountering the ENM [14]. This is further supported with evidence that the microtissues were shown to rotate within the wells, and thus ENM exposure is likely to be even across the surface of the spheroids [14]. Similarly, with the addition of an agarose coating at the base of the spheroids, the HepG2 spheroids are also free to move and rotate within the well enabling the ENMs to access the entire surface layer of actively proliferating HepG2 cells [28].

There was a significant difference in the genotoxicity observed between acute and prolonged ENM exposure, whereby the notable ENM-associated genotoxic effects observed in the first 24 h are not apparent over the prolonged exposure. This could be due to repair mechanisms that, may be efficient at removing DNA damage and / or damaged cells over time, which is not evident in an acute exposure experiment. Additionally, cells developing MN within the first 24 h of exposure can undergo cell death over the remaining duration of the prolonged experiment. It is also important to consider that following the prolonged exposure periods, although more individual cells were scored in the analysis, there is a greater chance of the DNA damage observed in the first 24 h to have been “diluted” in the ever-growing population. As a result, the probability of scoring a cell with a MN decreases over time. However, these time-dependent differences in genotoxicity could also be as result of extrinsic ENM physico-chemical properties (e.g. surface reactivity, agglomeration and dissolution). For example, ZnO was the only ENM to exhibit genotoxic effects following both acute and prolonged ENM exposure, with a significant response induced at 0.5–10.0 µg/mL in the acute exposures and 0.2–5.0 µg/mL in the prolonged exposures. ZnO nanoparticles are known to be highly soluble and readily release ions [61]. Aligned with existing literature, the ZnO ENMs in this study were found to rapidly dissolve into Zn^2+^ ions and two hydroxide ions, the latter causing an increase in pH in the culture medium within 15 min of exposure [37]. For soluble metal ENMs, there is always a question as to whether the toxicity observed is caused directly by the ENMs themselves or from the dissolved ions. ZnO ENMs can dissolve into Zn^2+^ ions, which can trigger signaling cascades leading to an enhanced influx of calcium, the release of (pro-)inflammatory mediators and ROS generation [40]. Interestingly, the greater induction of genotoxicity was observed following 24 h exposure to ZnO ENMs as opposed to 120 h. This is hypothesized to be induced by the rapid dissolution of Zn^2+^ ions within the first 24 h, inducing acute adverse effects similar to those described above, leading to an elevated genotoxic effect which was no longer as prominent following 120 h exposure, as a result of the reduced number of ions released over the 120 h exposure period. This correlates with reports in the literature whereby, an acute 24 h ZnO (NM-111) exposure caused a loss in the glutathione levels and an increase in the levels of ROS in the human hepatoblastoma C3A cell line [62]. Further to this, by employing the anti-oxidant Trolox, a reduction in IL-8 response and a suppression in the toxicity potential of the ZnO ENMs was observed, thus highlighting the important link of ZnO mediated oxidative stress. This elevated ZnO induced oxidative stress and ROS, has been shown to cause oxidative DNA damage, including DNA strand breaks and formamidopyrimidine DNA glycosylase (fpg)-specific DNA lesions in the liver [63, 64]. In addition, there are concerns that even if ZnO ENMs were not able to enter the nucleus, the Zn^2+^ ions could interact and affect DNA integrity, making successful DNA repair even more challenging [40]. These reports in the scientific literature indicate that persistent release and accumulation of ions over time could further induce ongoing oxidative stress and ROS induced DNA damage, as well as impeding DNA repair mechanisms, resulting in a prolonged genotoxic effect, similar to that found in this study.

Although Ag ENMs release Ag^+^ ions in a similar manner to ZnO, the Ag were found to be the least genotoxic out of the five materials tested. It is possible these Ag^+^ ions may also induce DNA damage, but they are released much more slowly than the Zn^2+^ ions; thus, it is hypothesized that the gradual dissolution of Ag ENMs over the 24 h period could allow time for the repair of any low level damage induced. Additionally, the Ag ENMs were found to exhibit substantial dose-dependent agglomeration, with almost a 12-fold increase in the average particle diameter between the lowest and the highest concentrations, which could restrict the number of Ag particles internalized by the hepatocytes. Any Ag ENMs not internalized by the cells would remain within the culture medium and subsequently dissolve into Ag ions in situ, where the ions would likely be sequestered by the excess serum proteins in the medium. Consequently, the extrinsic physico-chemical variances between ZnO and Ag ENM are likely to account for the differences in their ability to induce genotoxicity.

Due to the more prominent genotoxic nature of ZnO and TiO_2_, these two materials were taken forward for further assessment into the effects different prolonged exposure regimes may have upon the genotoxic potential of ENMs. It was important to test both materials, as ZnO was known to give a positive genotoxic response following both acute and prolonged exposures, whilst TiO_2_ only induced genotoxicity after an acute exposure. Interestingly, the enhanced complexity of the repeated exposure regime, developed to better mimic the natural human exposure scenario, made no difference on the toxicological response (cytotoxicity, (pro-)inflammatory response nor genotoxicity) in the HepG2 liver spheroids. A previous study, using the same materials, ZnO (JRC NM-111) and TiO_2_ (JRC NM-105), showed that a repeated exposure of 0.62–10.0 µg/mL dosed every other day for up to 21 days induced limited cytotoxicity, but a time dependent increase in cytokine levels in PHH microtissues [14]. Kermanizadeh et al., however, did not fractionate the doses over this time period and also included a 24 h recovery period between doses and an extended recovery (≥ 7 days) at the end of the exposure duration, which was shown to help alleviate the (pro-)inflammatory response. In that study, whilst an almost complete refresh of the culture medium was undertaken between repeated exposures, as the doses were not fractionated the final concentration of the ENM exposures will have been greater than those used in the present study as a result of residual material. Consequently, even with a repeated ENM exposure regime that is four-fold longer than the one described in the present study, neither TiO_2_ or ZnO induced any adverse effects in either hepatic model. This could be explained through the liver’s unique ability to regenerate itself following acute toxicological insult, and with the low doses used for the repeated exposure in this study, toxicity may have been within limits that did not overwhelm the hepatocytes nor induce adverse outcomes. Another aspect to consider is whether the added complexity of a prolonged, repeated exposure outweighs the benefits of a more simplistic dosing approach. In the present study, the added complexity of repeated, fractionated exposures did not improve the predictive capabilities of the in vitro 3D liver model when evaluating ZnO and TiO_2_. Thus, while prolonged repeated ENM exposures performed with in vivo relevant concentrations are more physiologically relevant and provide a better insight into the long-term effects of ENM exposure upon the liver, fractionated, repeated dosing regimens may not provide additional benefit for assessing the toxicological response in hepatocytes over single, bolus dosing regimens.

## Conclusion

In conclusion, both acute and prolonged ENM exposures were assessed and were shown to result in different toxicological responses. This highlights the importance of evaluating prolonged ENM exposures to fully understand the longer-term and accumulated effects of ENMs following acute insult. For ZnO and TiO_2_, there was no significant difference between prolonged single, bolus or repeated, fractionated exposure regimes. Thus, the added complexity of fractionated dosing did not influence the study outcome. Even given the low doses of ENM applied in this study, all five of the materials tested were shown to induce fixed DNA damage in 3D HepG2 spheroids following acute exposure, leading to the following genotoxicity potency ranking: ZnO > TiO_2_ > BaSO_4_ = CeO_2_ > Ag. This study therefore demonstrates that 3D in vitro hepatic spheroid models have the capacity to be utilised for evaluating more realistic ENM exposures, thereby providing a future in vitro approach to better support ENM hazard assessment in a routine and easily accessible manner.

## Materials and methods

### In vitro 3D liver model

The Human Caucasian Hepatocellular Carcinoma derived epithelial cell line, HepG2 (ECACC 85011430 and ATCC HB-8065) monolayers were cultured in Dubecco’s Modified Eagle Medium (DMEM) with 4.5g/L d-Glucose and L-Glutamine (GIBCO, Paisley, UK) supplemented with 10% Foetal Bovine Serum (FBS) and 1% Penicillin/Streptomycin antibiotic (GIBCO, Paisley, UK). HepG2 cells were sub-cultured every 3–5 days, once 80% confluency was reached, they were trypsinised (0.05% trypsin/EDTA solution; GIBCO, Paisley, UK) and a cell stock of 2.0 × 10^5^ cells/mL was prepared. To form the 3D spheroid structure, HepG2 cells were cultured in 96-well plates using the hanging drop method developed by Llewellyn et al. and described by Conway et al. [28, 29]. Extensive information regarding the establishment, culture, characterisation, exposure protocol, harvest and application of the 3D HepG2 spheroid model can be found in the published protocol by Llewellyn et al., 2020. In short, 20 μL of the cell suspension (4000 HepG2 cells per 20 μL hanging drop) was pipetted onto the inverted side of a 96-well tissue culture plate, before gently inverting the lid and placing back onto the 96-well plate filled with 100µl of PBS [28]. The plate was then placed in the incubator at 37 °C with 5% CO_2_ for 3 days before agarose transfer.

### ENM characterisation

#### Crystalline phases (XRD)

Powder samples were loaded onto a 20 × 20 mm glass sample holder, and the incident X-rays aligned to enter the centre of the sample. The X-ray is generated by a rotating anode X-ray generator of Copper (Cu). We executed a 2θ−θ coupled scan from 10 °C to 100 °C with a step width of 0.02 °C and a second duration time per step. Measured data is refined by Rietveld analysis using PDXL from Rigaku SmartLab. XRD analysis were performed in line with ISO 17025 and, technically to, JIS K 0131 and BS EN 13925-4 [65–67].

#### Impurities (XRF)

Semi-quantitative elemental analysis was conducted on powder samples of the ENMs using a Bruker S8 Tiger wave length dispersive X-ray fluorescence (WDXRF) spectrometer using Rh X-ray source operated at 60 kV. Powdered samples of 2.0–5.0 mg were placed on a XRF thin film (mylar sheet with a thickness of 6.0 µm), which was fixed in a 40 mm diameter sample cup (Fluxana, Kleve, Germany). The measurement time was 17 min. Results were manually post-processed for each element individually, to account for low concentrations and peak overlaps.

#### TEM (size, 3D aspect ratio and circulatory)

TEM was carried out for ENMs on an EM208, operating at 200 kV (Philips, Eindhoven, The Netherlands), with a high definition acquisition system based on a side-mounted TEM camera OSIS Morada and an iTEM soft-ware platform (Olympus Soft Imaging Solutions GmbH, Münster, Germany). ENMs, dispersed in MilliQ water, were placed onto a carbon-coated grid and dried at room temperature under vacuum.

#### Surface area (BET)

Specific surface area was determined with the BET method using a Micromeritics Gemini V. Samples were degassed at 100 °C under vacuum for 30 mins. Nitrogen adsorption isotherms at 77 K were recorded at five pressures between 0.05 and 0.30 P/P0. Measurements were performed adhering to the standard DIN ISO 9277-2014-01 [68].

#### Density (He pycnometer)

Skeletal density of all ENMs was determined using a He pycnometer (Micromeritics AccuPyc II 1340). Samples were measured at 20 °C, applying ten He purging cycles of the chamber before the measurement and analyzed according to DIN EN ISO 1183-3 [69].

#### XPS

XPS measurements were performed with a VersaProbe II Spectrometer (Ulvac-Phi, Japan) to obtain the chemical composition. The instrument was calibrated with clean Gold (Au) and Cu foils, of which electron binding energies were Au 4f=83.96 ± 0.02 eV and Cu 2p_3/2_= 932.62 ± 0.05 eV, respectively [70]. The samples were irradiated with monochromatic Al Kα X-ray (ħω=1486.6 eV, 25 W) using an X-ray spot size of 100 × 100 μm^2^ and a take-off angle of 45 º with respect to the sample surface. The base pressure of the instrument was better than 1.0 × 10^-9^ Torr and the operating pressure better than 3.0 × 10^-9^ Torr. The surface chemical compositions (as %) were determined by relative atomic sensitivity factors. The samples were not etched or pre-treated prior to each measurement.

#### Surface charge

The zeta potential (ZP) was measured at room temperature (25 °C) as a function of pH using a ZP analyzer (Malvern Zetasizer Nano ZS). Each ZP value was calculated in an average of 22–30 runs at pH 7 in 10 mM potassium chloride (KCl) water solution.

#### FRAS

For ENM reactivity testing under physiological conditions, the FRAS assay multi-dose protocol was undertaken as described by Gandon et al. [71].

#### EPR spin trap DMPO and CPH

Two standardized EPR methods have been established to assess the surface-induced reactivity of ENMs: method I utilizes the nitrone spin trap DMPO, one of the most established spin traps for nanosafety purposes, whilst method II employs the cyclic hydroxylamine spin probe CPH which interacts directly with short-lived ROS (e.g*.* superoxide radical) on the material surface [72, 73].

#### Hydrophobicity

The material hydrophilicity was evaluated by a water contact angle measurement using Drop Shape Analyzer - DSA100. Sample powder (~ 0.5 g) was spread as a thin layer on the surface of the sticky sample holder (3M Color Laser Transparency Film plate covered with a homogenous adhesive layer (0.25 mm) of Acronal^®^ V 215) by pressing the surface with a spatula. A nitrogen gun is used to gently blow the powder residuals not attached to the sample holder’s surface. Finally, contact angle measurement was performed at 23 °C by measuring the diameter of the spherical crown of 2 μL water dropped on the surface of sample layer.

#### Dynamic light scattering (DLS)/electrophoretic light scattering (ELS) measurements

The colloidal characterization of TiO_2_, ZnO, Ag, BaSO_4_ and CeO_2_ ENMs was determined using a Zetasizer nano ZSP (model ZEN5600, Malvern Instruments, UK), measuring the DLS (ØDLS) and ZP of nanosol. ZP measurements were performed by ELS and the Smoluchowski equation was applied to convert the electrophoretic mobility to ZP. ENMs were diluted at 0.2, 0.5, 1.0, 2.0, 5.0 and 10.0 µg/mL in DMEM complete for DLS and ZP analysis and measured after 24 and 120 h of exposure at 37 °C in static condition. Samples were measured three times and the mean ØDLS and ZP data presented.

#### Sensor dish reader reactivity and Dissolution testing

Real-time temporal pH and O_2_ reactivity and 24 h end-point dissolution testing was performed using the Sensor Dish^®^ reader (SDR) method (PreSens Precision Sensing GmbH, Regensburg, Germany). The test is based on the use of fluorescent pH (HydroDish™; range: pH 6–8.5, resolution 0.05 pH units) and O_2_ (OxoDish™; range: 0–50% dissolved O_2_; measured in mmol/L) sensors mounted at the bottom of each well in 24-well multi-dish cell incubation plates. The tests were conducted in DMEM + 10% FBS + 1% Pen/Strep, similar to the medium used in the in vitro assays. A standard material concentration of 320 µg/mL was used to obtain sufficiently robust reactivity signal from the test materials as compared with the reactivity signal from the pure medium.

Batch-dispersions of 2.56 mg/mL were made in 0.05% BSA-water by 16 min, 13 mm probe-sonication, amplitude 10% (Branson Sonifier S-450D, Branson Ultrasonics Corp., Danbury, CT, USA) after pre-wetting the test materials with ethanol following the NANOGENOTOX dispersion protocol [74] and added by pipette to the test media immediately after dispersion was completed.

For each test, SensorDish^®^ plates with 1.750 mL test medium added to each well were placed on the plate readers in a cell incubation chamber (37 °C; 5% CO_2_ atmosphere; 95% Relative humidity; CelCulture^®^ CO_2_ Incubator, ESCO Medical, Egaa, Denmark). After thermal equilibration to 37 °C, a batch dispersion was prepared for the test material in question. 250 µL batch dispersion or control dispersion medium were each added to half of the wells, respectively and online measurement of the pH and O_2_ concentrations was started immediately. After 24 h the measurements were stopped and medium samples were collected and added to 3 kDa centrifugal filter tubes by pipette and centrifuged at 4000 × RCF for 30 min. The 3 kDa filtered medium were sampled and added 500 µL 2% ultrapure HNO_3_. The amount of liquids were weighed for subsequent quantification. Liquid samples were stored in darkness until shipment for inductively coupled plasma mass spectrometry (ICP-MS) analysis. The temporal reactivities (dpH and dO_2_) were calculated as the difference between the mean values in wells with a test material minus the mean values in wells without a test material and then plotted as function of time. The SDR-test is explained in detail in Jørgensen et al. (in prep.)

### ENM exposures

Five ENMs (TiO_2_ NM-105, ZnO NM-111, JRC Nanomaterials Repository, Belgium; BaSO_4_ NM-220_,_ CeO_2_ NM-212, Fraunhofer IME, Germany; and Ag 576832, Sigma Aldrich, UK) were stored as dry powders at room temperature until the day of exposure. ENM stock solutions were prepared (2.56 mg/mL) and dispersed for 16 mins in 0.05% Bovine Serum Albumin (BSA) using the probe sonication (Branson Sonifier 250, Ø 13 mm, 400 W output power, 20 kHz) method described by Jensen et al. [74]. Working stocks of ENMs were made fresh for each experiment. Following dispersion, ENMs were diluted in cell culture media to the required concentrations with all five ENMs assessed over both an acute 24 h and prolonged 120 h exposure scheme. For prolonged ENM exposures, a partial media change was undertaken after 72 h, whereby the top 50% of the culture medium within the wells was removed and replaced with fresh ENM-free culture medium of the exact same volume. Exposure procedures are described in detail, with a peer-reviewed SOP available, in Llewellyn et al. [29]. TiO_2_ and ZnO at two selected doses of 0.5 and 5.0 µg/mL, were further evaluated following a longer-term (120 h), repeated dosing scheme whereby the original bolus dose was fractionated into five, equal parts of 0.1 and 1.0 µg/mL, respectively to be dosed daily onto the 3D liver spheroids, Fig. 4. The plates were then incubated at 37 °C/5% CO_2_ for the desired exposure period. For prolonged exposures, a culture medium replacement was undertaken once, on Day 3 of the exposure, by removing 50 µL of media from the well and replacing with a fresh 50 µL of DMEM. For the repeated ENM exposures however, this was not necessary, as 50% of the culture medium was being refreshed daily with the new dose of ENMs. All experiments were performed with three biological replicates with mean data ± SD presented, unless stated otherwise.

### Liver functionality: albumin and urea assays

Following both acute and prolonged ENM exposure, liver-like functionality was evaluated using the BCG Albumin Assay Kit (MAK124, Sigma, UK) and Urea Assay Kit (MAK006, Sigma-Aldrich, UK). A negative, untreated media control was used alongside a chemical positive control; 0.1 µM of a known liver carcinogen, Aflatoxin B1 (Afla B1; Cat# No: A6636, Sigma Aldrich, UK). At the end of the exposure period all supernatants were harvested by pooling 50 µL of media from each well. To sediment any excess ENM from the supernatant, the samples were centrifuged at 230*g* for 2 mins and the resulting supernatant collected. All assays were performed as per manufacturer’s instructions, with three biological replicates assessed in triplicate. For the urea assay, supernatants were diluted 1:10 with urea assay buffer.

### (Pro)-inflammatory response: interleukin-6 (IL-6), interleukin 8 (IL-8) and tumour necrosis factor alpha (TNF-α)

Cytokine release was quantified by ELISA, using the cell supernatants described above. DuoSet human antibody kits for IL-8, IL-6, and TNF-α (DY208, DY206 and DY210 DuoSet ELISA, R&D Systems) were used according to the manufacturer’s instructions. An ELISA assay positive control, Tumor Necrosis Factor Alpha Protein (TNF-α protein; Cat# No: 2-35076, BioTechne, UK) was re-suspended in ddH_2_O according to manufacturer’s instructions and diluted to a final working concentration of 0.25µg/mL of TNF-α protein. The detection antibodies were diluted as follows: IL-8: 0.1% BSA, 0.05% Tween 20 in Tris-buffered Saline (TBS) and IL-6/TNF-α: 1% BSA in PBS, and incubated with samples for 2 h at RT. The signal was developed using streptavidin horseradish-peroxidase and TMB Substrate Reagent A and B (Cat# No. DY999, R&D Systems, UK). Absorbance was measured at 450 nm (PolarStar Omega Plate Reader) and the standard curve was plotted as 4-parameter logistic fit using the MyAssays.com software. Three biological replicates were assessed in triplicate.

### Cytotoxicity and genotoxicity: trypan blue exclusion assay, cytokinesis proliferation index and in vitro micronucleus assay

The MN assay was undertaken in conjunction with the cytokinesis-block proliferation index (CPBI) and trypan blue exclusion assay for determining cytotoxicity post-acute and longer-term ENM exposures, respectively. A negative, untreated media control was used alongside 0.1 µM of a known liver carcinogen, Afla B1 as a positive control for genotoxicity. The MN assay was conducted as described by Conway et al. [28]. In short, after both acute and prolonged exposures, the cell culture supernatant was harvested and stored at – 80 °C for future biochemical endpoint analysis. The remaining liver spheroids were then pooled, trypsinised and prepared for cytotoxicity assessment and semi-automated MN scoring as previously described by Llewellyn et al. and Conway et al. [28, 29]. When scoring, detection of micronuclei in bi-nucleated or mono-nucleated cells were performed as previously described by Llewellyn et al. [29]. A minimum of 1000 bi-nucleated cells or 2000 mono-nucleated cells were counted per exposure dose per biological replicate (n ≥ 2), using the principles established by Fenech et al. and in accordance with the OECD Test No. 487: In Vitro Mammalian Cell Micronucleus Test guidelines [75, 76]. All controls for the MN assay were within the acceptance criteria based on historical ranges, with the average MN frequency for the positive control (Aflatoxin B1) lying between 2.2 and 2.8% and the negative, untreated control data between 0.8 and 1.4%. In all tests, the positive control had to be a minimum of two-fold greater than that of the untreated, negative control.

### Data analysis and statistics

Statistical analysis was performed using Prism 8, GraphPad Software, Inc. (USA). Shapiro-Wilk test was used to calculate normality for each data set. For normally distributed data, One-way ANOVA with Sidak’s post hoc were used. For non-parametric data, Kruskal-Wallis test was used to calculate significance when there were more than two variables, with Dunn’s multiple comparisons test. For genotoxicity data sets, with ≥ 2 biological replicates, a two-tailed Fischer’s Exact test was conducted.

## Supplementary Information

**Additional file 1:** **Figure S1**: TEM micrographs of the ENM listed in Table 1: (**A**) TiO_2_ NM-105 (**B**) Ag Sigma (**C**) BaSO_4_ NM-220 (**D**) CeO_2_ NM-212 and (**E**) ZnO NM-111. Image (**A**), (**B**), (**C**) and (**D**) reproduced from Keller et al. (2020) (https://doi.org/10.1080/17435390.2020.1836281) and image (**E**) reproduced from Yin et al. (2015) (https://doi.org/10.1007/s11051-014-2851-y). **Figure S2:** A series of XRD patterns for the five ENMs listed in Table 1, (**A**) TiO_2_ (NM-105), (**B**) Ag (Sigma 576832), (**C**) ZnO (NM-111), (**D**) BaSO_4_ (NM-220) and (**E**) CeO_2_ (NM-212). These graphs illustrate the crystalline phases for each material as summarised in Table 1. **Figure S3: **A series of XPS core level curves for the five ENMs included in this study and summarized in Table 1: TiO_2_ (NM-105), ZnO (NM-111), Ag (Sigma 576832), BaSO_4_ (NM-220) and CeO_2_ (NM-212). Each curve is fitted by Lorentzian-Gaussian convoluted functions to determine the chemical composition. **Figure S4: **Representative images of micronuclei generated by automated scoring of HepG2 cells using a Metafer MetaSystem 3.9.8. (**A**) illustrates an enlarged image of a micronucleus shown in the scoring gallery pictured in (**D**), and highlighted with an orange outline. Representative images of micronuclei found within the HepG2 mononucleate (**B, C**) and binucleate (**E–G**) cell populations following prolonged and acute ENM exposures respectively.

## Data Availability

The datasets generated and analysed during the current study are uploaded to eNanoMapper database repository [https://search.data.enanomapper.net/projects/patrols].
