# Supplementary material for: Understanding the impact of more realistic low-dose, prolonged engineered nanomaterial exposure on genotoxicity using 3D models of the human liver
Source: J Nanobiotechnology. 2021 Jun 28;19:193. doi: 10.1186/s12951-021-00938-w (PMC8240362; doi:10.1186/s12951-021-00938-w)
Supplement: Supplementary file 1 — Additional file 1: Figure S1: TEM micrographs of the ENM listed in Table 1: (A) TiO2 NM-105 (B) Ag Sigma (C) BaSO4 NM-220 (D) CeO2 NM-212 and (E) ZnO NM-111. Image (A), (B), (C) and (D) reproduced from Keller et al. (2020) (https://doi.org/10.1080/17435390.2020.1836281) and image (E) reproduced from Yin et al. (2015) (https://doi.org/10.1007/s11051-014-2851-y). Figure S2: A series of XRD patterns for the five ENMs listed in Table 1, (A) TiO2 (NM-105), (B) Ag (Sigma 576832), (C) ZnO (NM-111), (D) BaSO4 (NM-220) and (E) CeO2 (NM-212). These graphs illustrate the crystalline phases for each material as summarised in Table 1. Figure S3: A series of XPS core level curves for the five ENMs included in this study and summarized in Table 1: TiO2 (NM-105), ZnO (NM-111), Ag (Sigma 576832), BaSO4 (NM-220) and CeO2 (NM-212). Each curve is fitted by Lorentzian-Gaussian convoluted functions to determine the chemical composition. Figure S4: Representative images of micronuclei generated by automated scoring of HepG2 cells using a Metafer MetaSystem 3.9.8. (A) illustrates an enlarged image of a micronucleus shown in the scoring gallery pictured in (D), and highlighted with an orange outline. Representative images of micronuclei found within the HepG2 mononucleate (B, C) and binucleate (E–G) cell populations following prolonged and acute ENM exposures respectively. [file 12951_2021_938_MOESM1_ESM.docx]

**Additional file 1**

1.
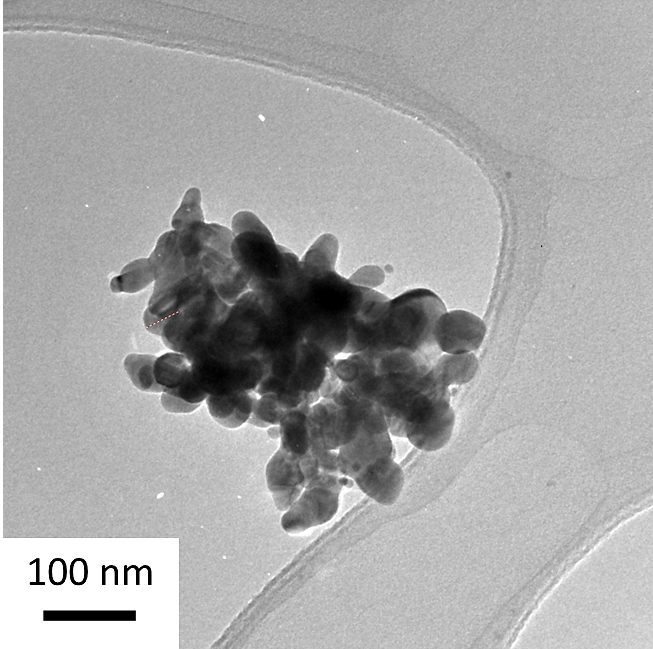
**ENM Characterisation**

**
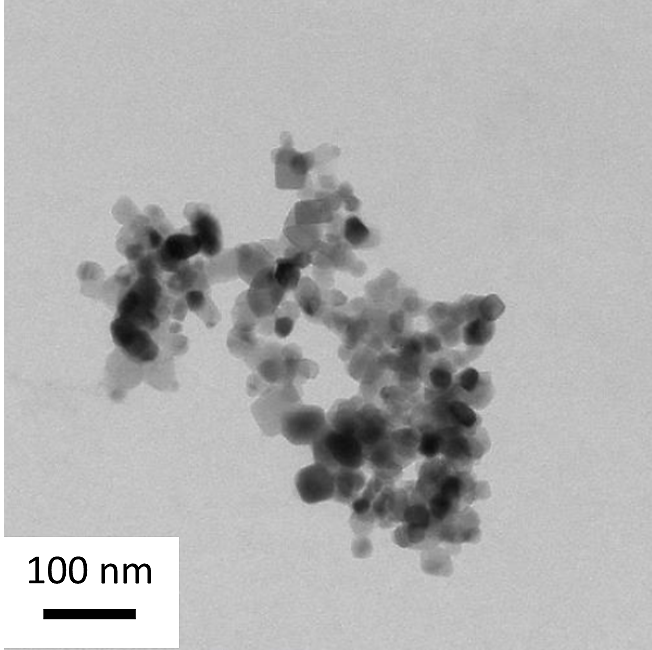
**

**B**

**A**

**NM-105**

**Sigma-576832**

**D**

**C**


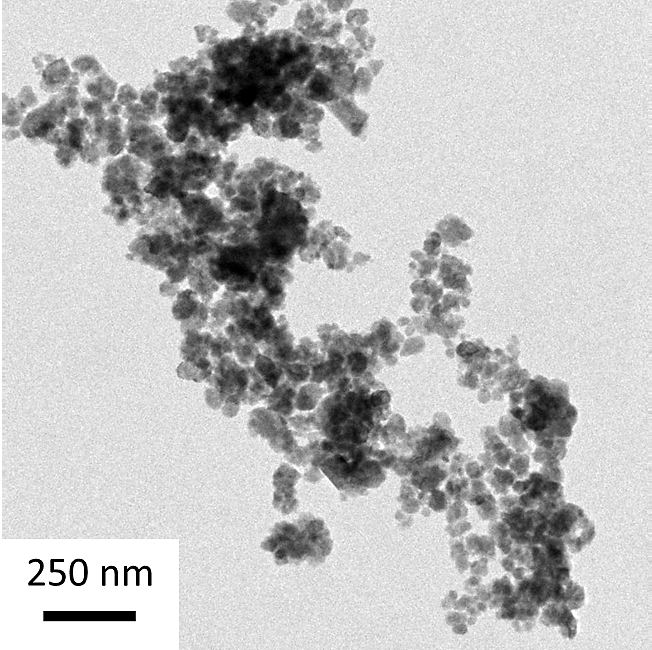


**NM-220**


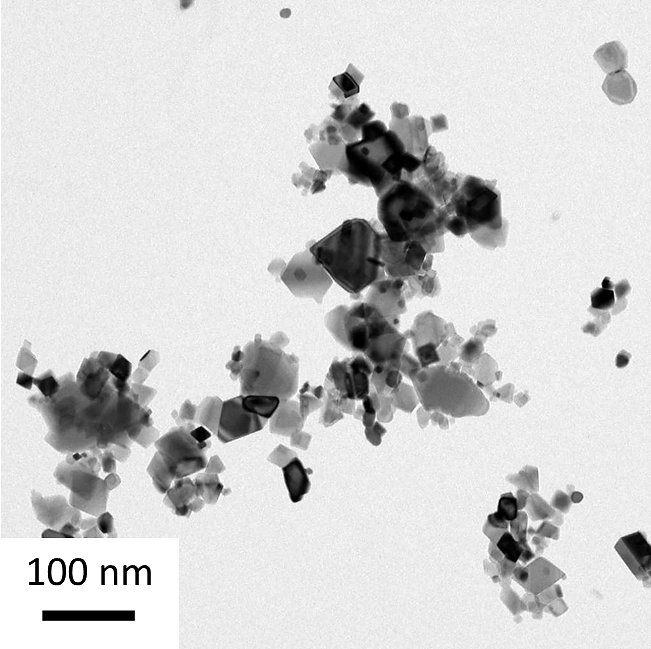


**NM-212**

**
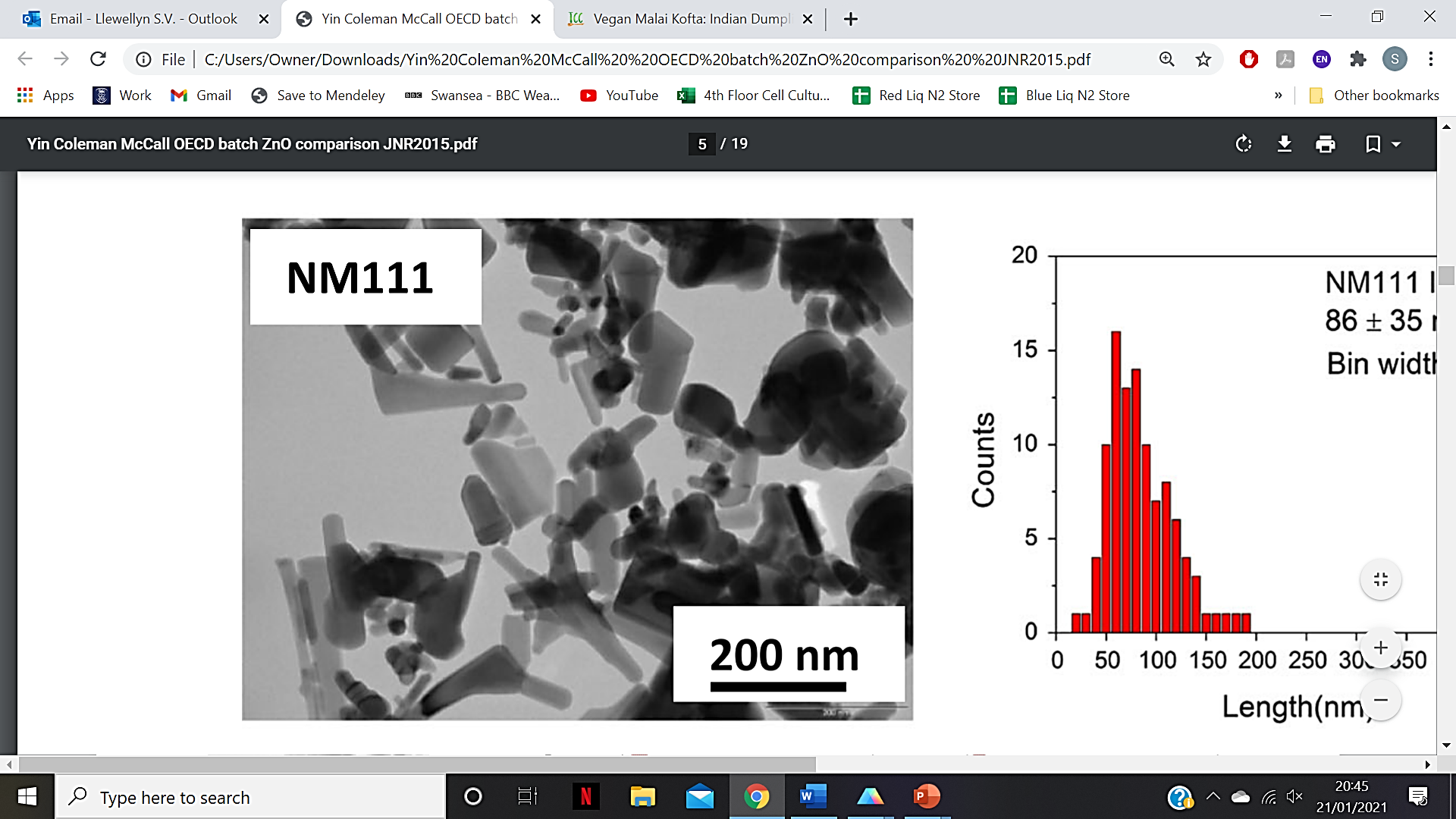
**

**E**

**Figure S1**: TEM micrographs of the ENM listed in Table 1: (A) TiO_2_ NM-105 (B) Ag Sigma (C) BaSO_4_ NM-220 (D) CeO_2_ NM-212 and (E) ZnO NM-111. Image (A), (B), (C) and (D) reproduced from Keller et al. (2020) (DOI: 10.1080/17435390.2020.1836281) and image (E) reproduced from Yin et al. (2015) (DOI 10.1007/s11051-014-2851-y).


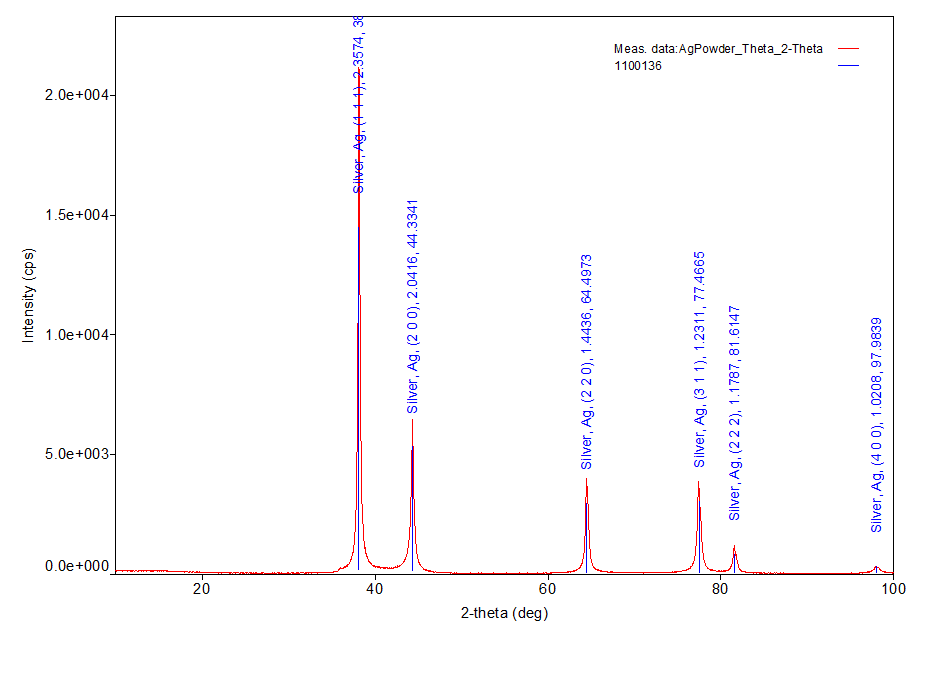


**B.** Ag Sigma-576832


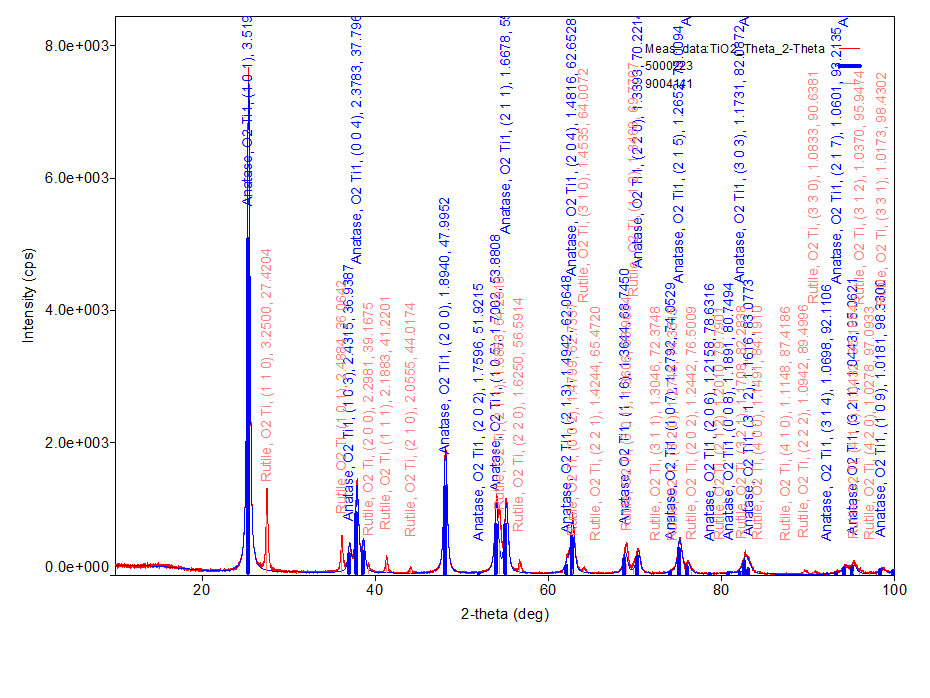


1. TiO_2_ NM-105

**C.** ZnO NM-111


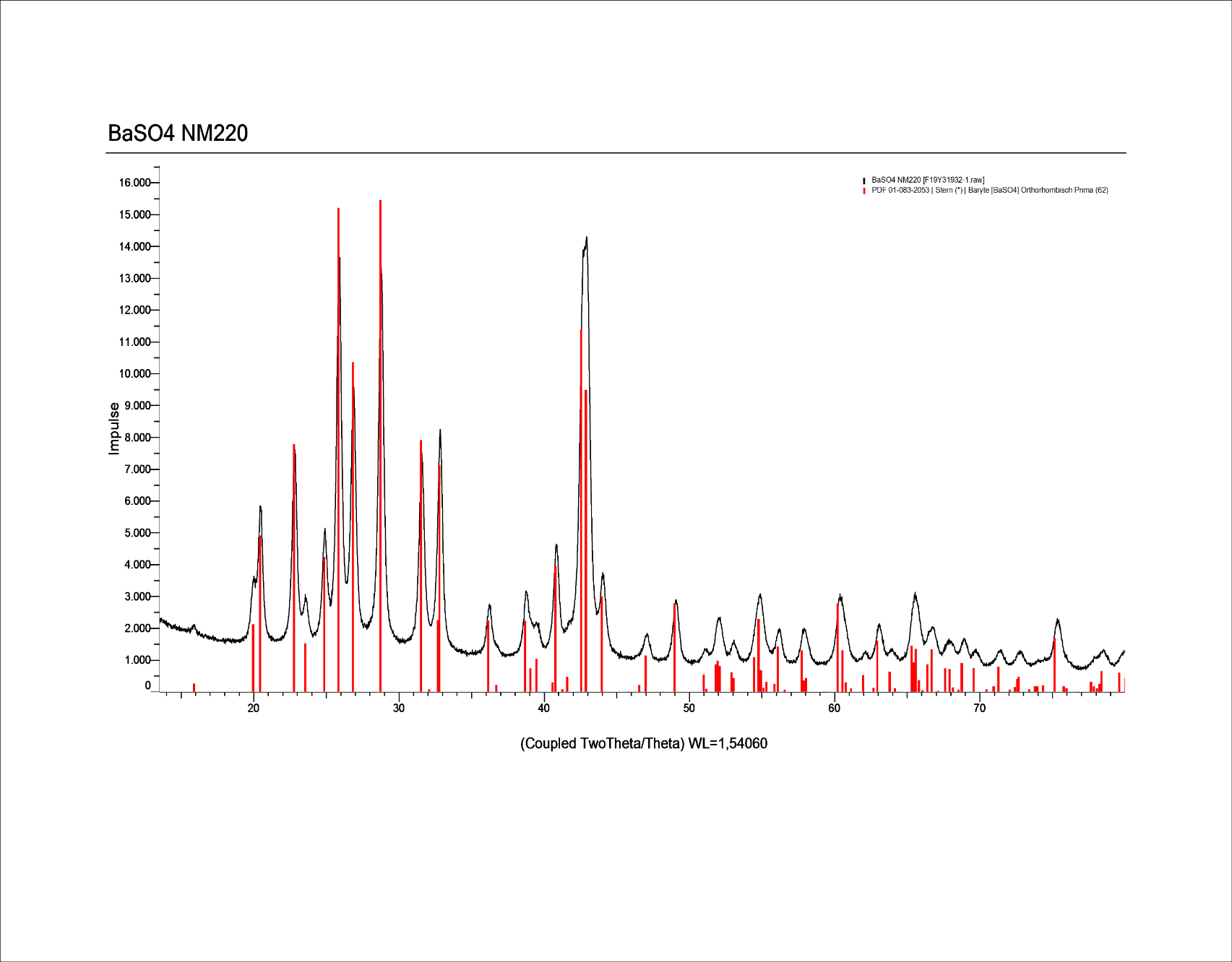


**D.**


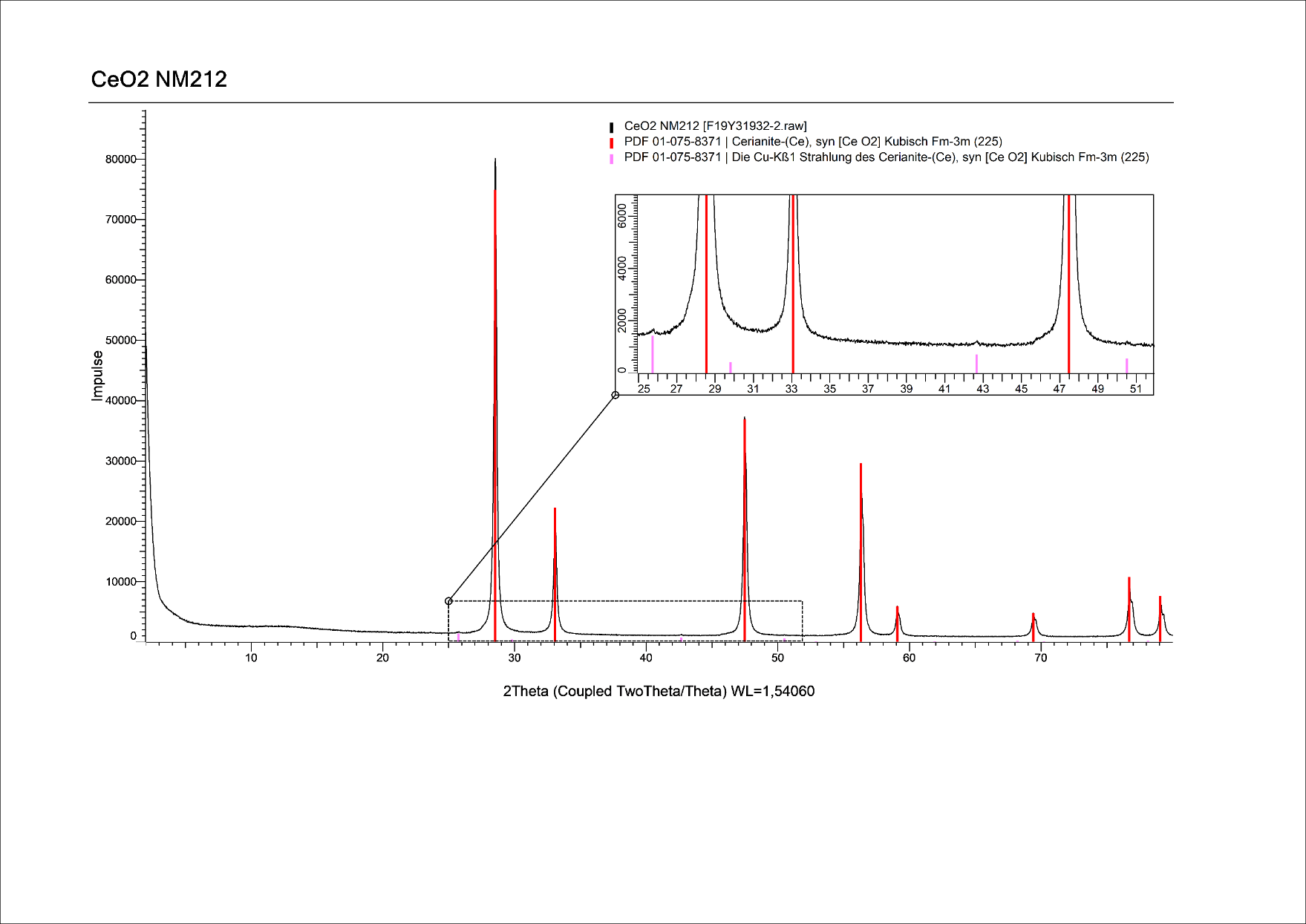


**E.**

**Figure S2:** A series of XRD patterns for the five ENMs listed in Table 1, (A) TiO_2_ (NM-105), (B) Ag (Sigma 576832), (C) ZnO (NM-111), (D) BaSO_4_ (NM-220) and (E) CeO_2_ (NM-212). These graphs illustrate the crystalline phases for each material as summarised in Table 1.

| NM-105 O 1s C 1s Ti 2p |
| --- |
| 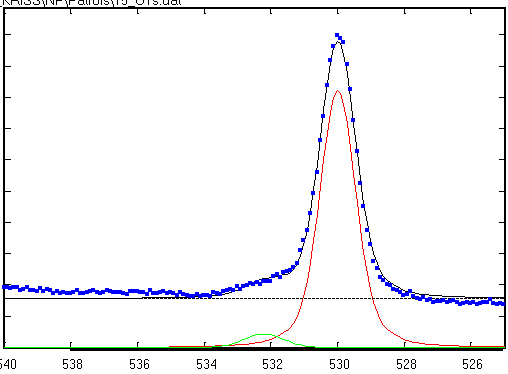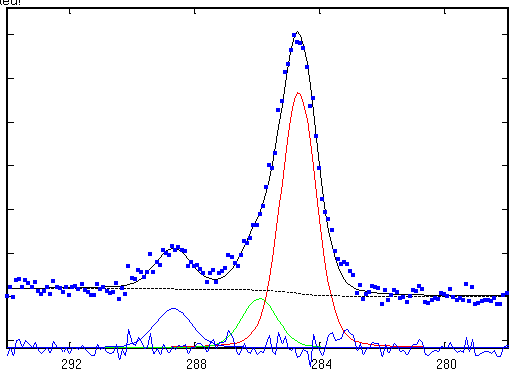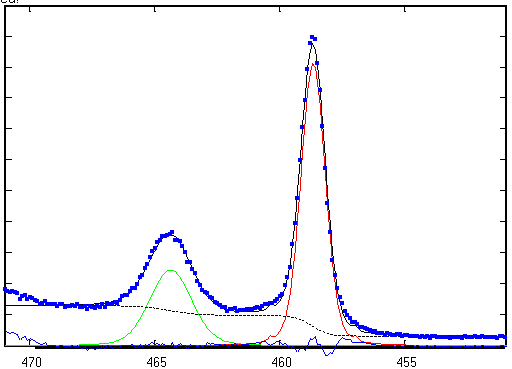 |
| NM-111 Zn 2p_3/2_ O 1s C 1s |
| 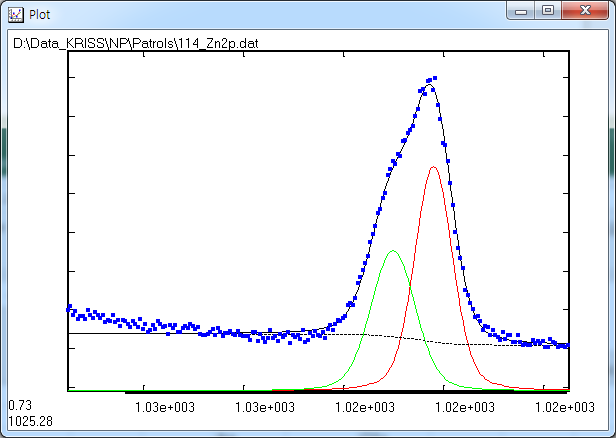 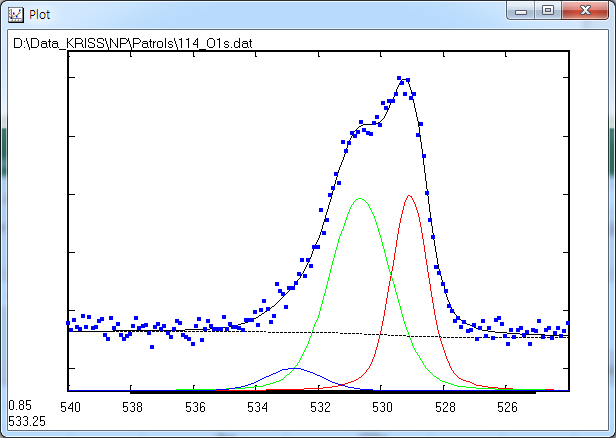 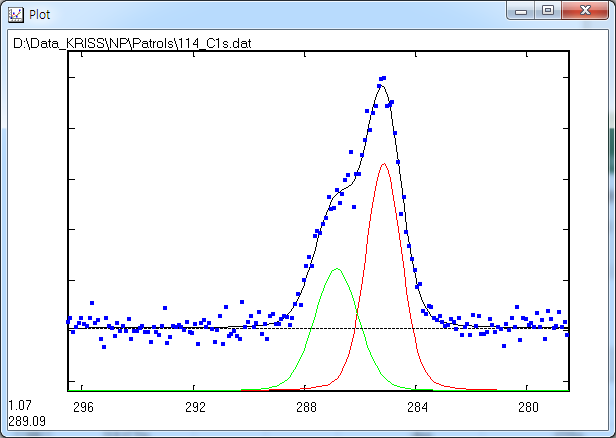 |
| Ag Sigma 576832 Ag 4d C 1s O 1s |
| 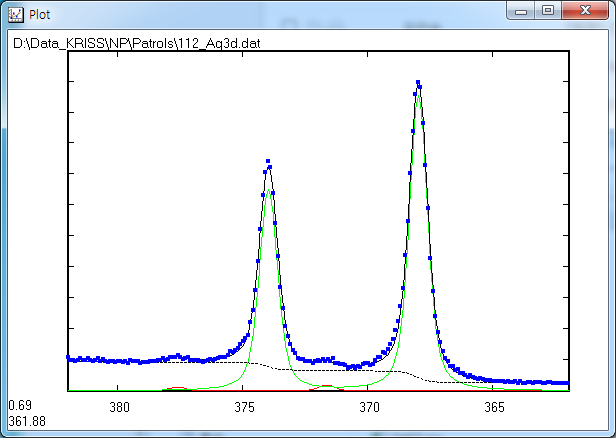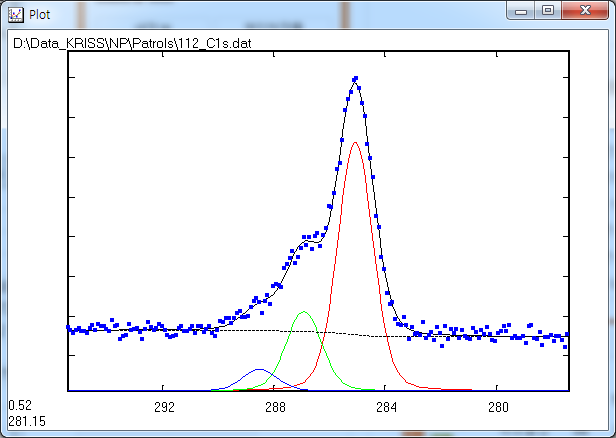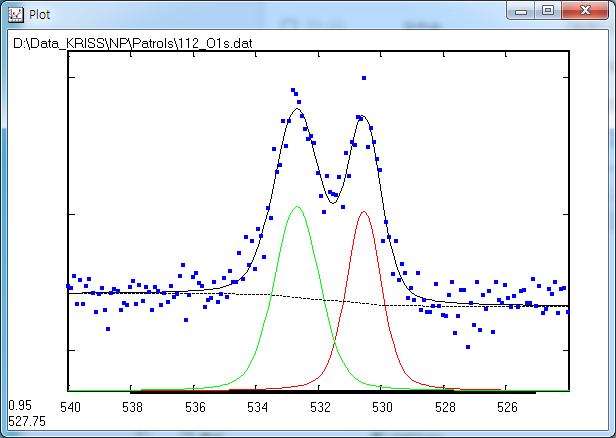 |
| NM-220 O 1s S 2p Ba 3d_5/2_ |
| 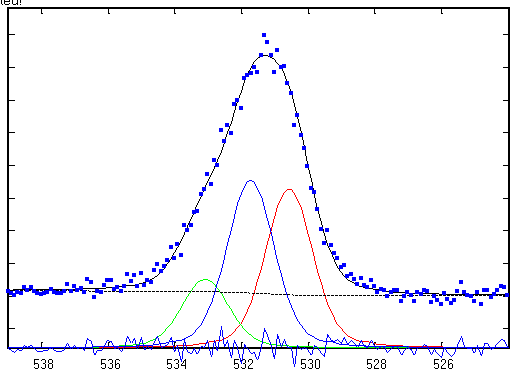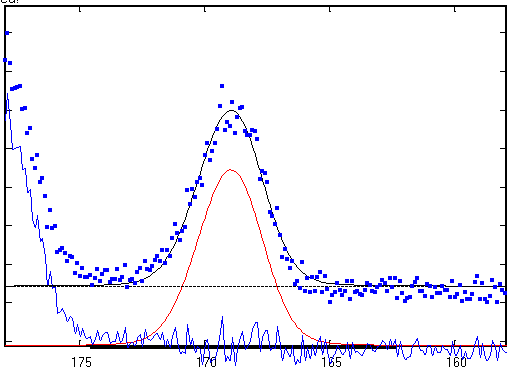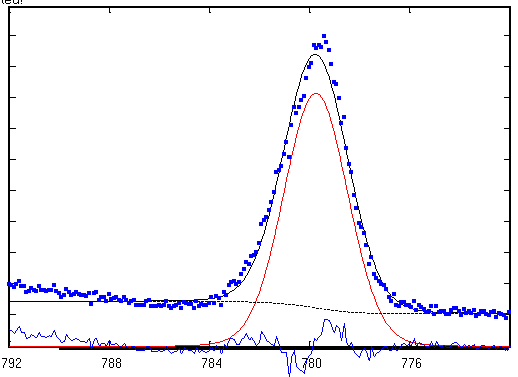 |
| NM-212 (q) Ce 3d (r) O 1s |
| 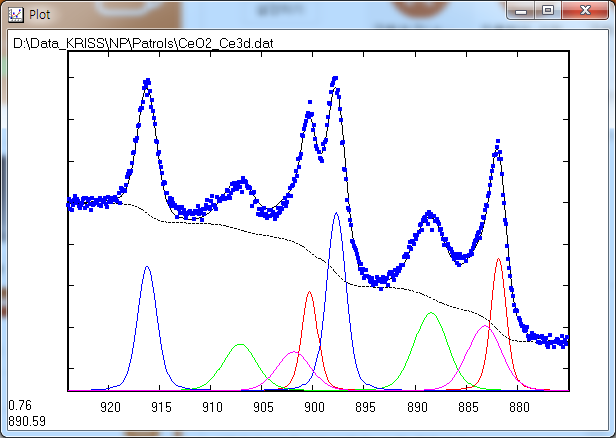 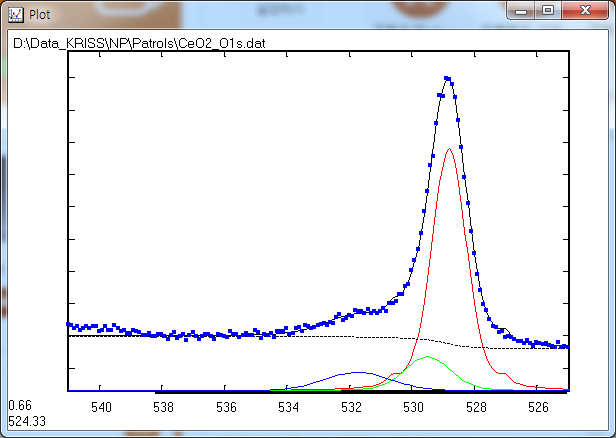 |

**Figure S3:** A series of XPS core level curves for the five ENMs included in this study and summarized in Table 1: TiO_2_ (NM-105), ZnO (NM-111), Ag (Sigma 576832), BaSO_4_ (NM-220) and CeO_2_ (NM-212). Each curve is fitted by Lorentzian-Gaussian convoluted functions to determine the chemical composition.

1. **Micronucleus Assay**


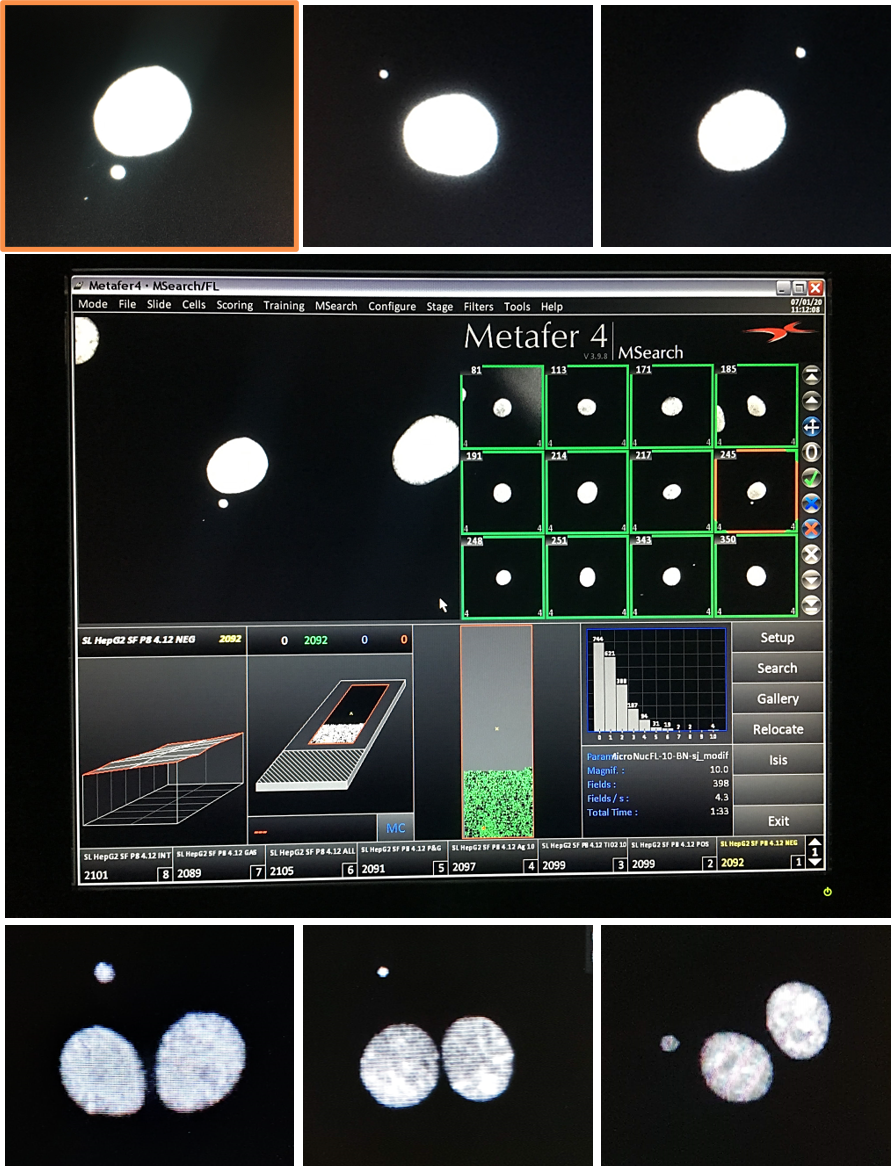


**A**

**B**

**C**

**D**

**F**

**E**

**G**

**D**

**Figure S4:** Representative images of micronuclei generated by automated scoring of HepG2 cells using a Metafer MetaSystem 3.9.8. (A) illustrates an enlarged image of a micronucleus shown in the scoring gallery pictured in (D), and highlighted with an orange outline. Representative images of micronuclei found within the HepG2 mononucleate (B, C) and binucleate (E – G) cell populations following prolonged and acute ENM exposures respectively.
